# Diagnostic and therapeutic pitfalls in the management of pediatric patients with 3β-hydroxysteroid dehydrogenase type 2 (3β-HSD2) deficiency - a single center experience

**DOI:** 10.3389/fendo.2025.1642051

**Published:** 2025-09-25

**Authors:** Zuzanna Gawlik, Ewelina Preizner-Rzucidło, Konrad Kaleta, Maria Szwarkowska, Martyna Wróblewska, Aleksandra Jurek, Teofila Książek, Krystian Jażdżewski, Anna Siejka, Jerzy Starzyk, Dominika Januś

**Affiliations:** ^1^ Department of Pediatric and Adolescent Endocrinology, University Children Hospital, Krakow, Poland; ^2^ Department of Pediatric and Adolescent Endocrinology, Chair of Pediatrics, Institute of Pediatrics, Jagiellonian University Medical College, Krakow, Poland; ^3^ Department of Medical Genetics, Chair of Pediatrics, Faculty of Medicine, Jagiellonian University Medical College, Krakow, Poland; ^4^ Students’ Scientific Group of Pediatric Auxology, Faculty of Medicine, Jagiellonian University Medical College, University Children’s Hospital, Krakow, Poland; ^5^ Laboratory of Cytogenetics and Molecular Genetics, University Children Hospital, Krakow, Poland; ^6^ Warsaw Genomics, University of Warsaw, Warsaw, Poland; ^7^ Clinical Biochemistry Department The Children’s Memorial Health Institute Al. Dzieci Polskich 20, Warsaw, Poland

**Keywords:** congenital adrenal hyperplasia, 3β-hydroxysteroid dehydrogenase deficiency, HSD3B2 gene, premature pubarche, ovarian cysts

## Abstract

**Introduction:**

Congenital adrenal hyperplasia (CAH) due to 3β-hydroxysteroid dehydrogenase type 2 deficiency (3β-HSD2D) is an exceptionally rare disorder affecting adrenal steroidogenesis, leading to variable clinical presentations. This study aims to highlight the phenotypic variability and management challenges associated with 3β-HSD2D through the analysis of three pediatric cases.

**Methods:**

We retrospectively reviewed three patients diagnosed with 3β-HSD2D at the Pediatric Endocrinology Department of the University Children’s Hospital in Krakow. Clinical features, laboratory findings, genetic analyses, and management strategies were evaluated. A detailed literature overview has been performed to find previously described 3β-HSD2D patients and correlate clinical presentation with distinct variants in the *HSD3B2* gene.

**Results:**

Case 1: A female neonate presented with adrenal insufficiency, electrolyte imbalances, hyperpigmentation, and congenital heart defects. Genetic testing revealed a homozygous missense pathogenic variant c.760T>G (p.Tyr254Asp) in the *HSD3B2* gene. Hydrocortisone and fludrocortisone therapy was introduced in the 2nd week of life. Case 2: A male infant exhibited atypical genitalia without salt-wasting crises. Compound heterozygous pathogenic variants c.760T>G (p.Tyr254Asp) and c.308-6G>A in *HSD3B2* gene were identified. He received therapy with testosterone prior to hypospadias correction and started therapy with hydrocortisone at the age of 1 y 10 m due to increased growth velocity and acceleration of bone age. Case 3: A female infant with salt-wasting crises and virilization was diagnosed with 3β-HSD2D. She additionally developed polycystic kidney disease, gallbladder stones and ovarian cysts. A pathogenic c.849del variant in homozygosity in *HSD3B2* was detected.

**Conclusions:**

This work underscores the clinical heterogeneity of 3β-HSD2D and the necessity for comprehensive genetic evaluation. Variants in the *HSD3B2* gene contribute to diverse phenotypes, complicating diagnosis and management. Retrospective evaluation of previously described cases offers us guidelines in the management of patients, who need multidisciplinary care involving endocrinology, genetic, gynecology, and urology specialists.

## 1 Introduction

Congenital adrenal hyperplasia (CAH) encompasses a group of genetic disorders characterized by impaired steroidogenesis (1). While most cases result from 21-hydroxylase deficiency (21OHD), accounting for over 90% of cases, rarer forms involving deficiencies of 11β-hydroxylase, 17α-hydroxylase/17,20-lyase, P450 oxidoreductase, steroidogenic acute regulatory protein (StAR), cholesterol side-chain cleavage enzyme (P450scc), and 3β-hydroxysteroid dehydrogenase type 2 (3β-HSD2D) have also been described (1–3).

3β-HSD2D is an exceptionally rare variant of CAH, caused by pathogenic variants in the *HSD3B2* gene, with an estimated incidence below 1 in 1,000,000 live births, representing approximately 0.5% of CAH cases (4–6). Two isoenzymes, 3β-HSD1 and 3β-HSD2, encoded by *HSD3B1* and *HSD3B2* respectively, share 93.6% sequence similarity and are located approximately 100 kb apart on chromosome 1p13.1, alongside five pseudogenes (7, 8). 3β-HSD1, expressed mainly in the placenta, breast, skin, liver, brain, and prostate, exhibits high substrate affinity and plays a vital role in placental progesterone production (9). In contrast, 3β-HSD2, predominantly expressed in the adrenal glands and gonads, mediates the rate-limiting step in steroid hormone synthesis, regulated by cortisol and sex steroids through feedback inhibition (9). It catalyzes the conversion of Δ5-3β-hydroxysteroids into Δ4-3-ketosteroids (9).

**Figure 1:**
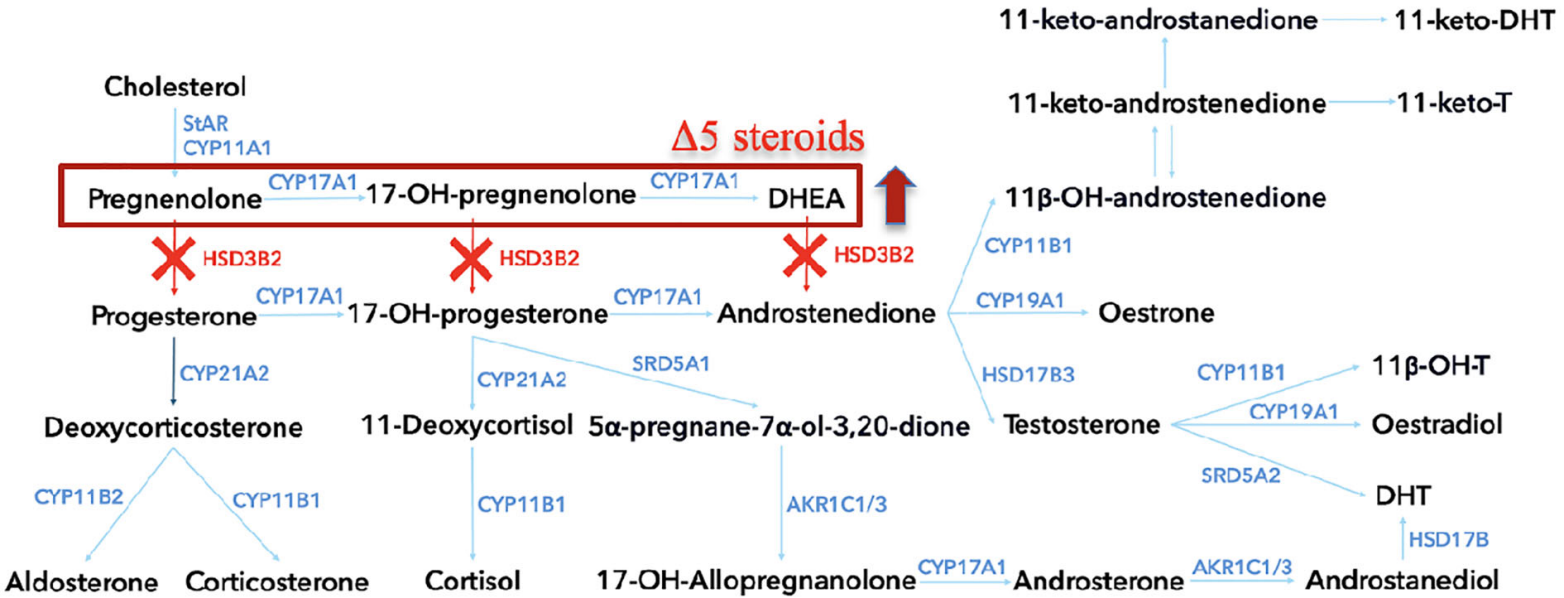
The steroidogenesis pathway, highlighting key enzymatic conversions involved in steroid hormone biosynthesis within the adrenal cortex. Each enzyme, represented by its gene symbol, is shown at the point of conversion it catalyzes. In cases of 3β-hydroxysteroid dehydrogenase type 2 deficiency, the pathway is disrupted at several points, as indicated by the red “X” marks. HSD3B2, 3β-hydroxysteroid dehydrogenase type 2; StAR, Steroidogenic acute regulatory protein; CYP11A1, Cholesterol side-chain cleavage enzyme; CYP17A1, 17α-hydroxylase/17,20-lyase; CYP21A2, 21-hydroxylase; CYP11B1, 11β-hydroxylase, CYP11B2, Aldosterone synthase; HSD17B3, 17β-hydroxysteroid dehydrogenase type 3; AKR1C1/3, Aldo-keto reductase family 1, member C1/3; SRD5A1, Steroid 5α-reductase type 1; SRD5A2, Steroid 5α-reductase type 2; HSD17B, 17β-hydroxysteroid dehydrogenase; DHEA, Dehydroepiandrosterone; 11-keto-DHT, 11-keto-dihydrotestosterone; 11-keto-T, 11-keto-testosterone; 11β-OH-T, 11β-hydroxytestosterone; 11β-OHT, 11β-hydroxytestosterone.

The *HSD3B2* gene, mapped to chromosome 1p12, contains four exons and three introns (10). Its protein product, comprising 371 amino acids, features essential functional regions: a cofactor-binding domain (residue 158) (11), a ligand-binding site (residue 154), two transmembrane segments, and a critical catalytic loop between L239 and Q251 (9, 12, 13). Mutations in *HSD3B2* impair adrenal and gonadal steroidogenesis, leading to the accumulation of Δ5 steroids and resulting in adrenal insufficiency and sex hormone dysregulation (**Figure 1**) (3, 14). Clinical severity depends largely on residual enzyme activity and compensatory function of 3β-HSD1 in peripheral tissues.

Classic 3β-HSD2D typically presents in neonates with adrenal insufficiency, salt-wasting syndrome (vomiting, dehydration), and genital abnormalities (15). Males may show incomplete masculinization, while females often display mild virilization. Management includes lifelong glucocorticoid and mineralocorticoid replacement, with surgical correction of genital anomalies as needed.

Non-classic 3β-HSD2D manifests with milder features, as residual enzyme activity prevents salt wasting and preserves normal genital development. However, patients may develop signs of androgen excess and hypogonadism later in life, such as hirsutism, acne, menstrual irregularities, and infertility (5, 16).

Due to its rarity, 3β-HSD2D is associated with considerable genetic heterogeneity. More than 200 genetically confirmed cases and over 95 distinct *HSD3B2* pathogenic variants have been identified to date (**Table 1**), encompassing missense, nonsense, frameshift, splicing mutations, and deletions. Missense variants are the most prevalent, while nonsense and frameshift mutations are typically linked to severe, classical phenotypes.

**Table 1:** List of pathogenic variants in the *HSD3B2* gene described previously in literature.

*First author. publication year [Ref.No.]*	*c.DNA acc to ref. NM_000198.4*	*Protein*	*Ethnicity (number of cases=236)*	*Homozygous / compound heterozygous*	*Clinical presentation*
*Aslaksen S.Shehab MA.2019 (* 44 *)*	c.15C > A	p.Cys5*	Norwegian (1)	homozygous	SW, 46,XX + clitoromegalia, hyperpigmentation of genitalia, premature ovarian insufficiency, autoimmune Addison's disease, sister died in an adrenal crisis at 2 y.o.
*Zhang L. 2000 (* 51 *)*	c.16C>T	p.Leu6Phe	Pakistani (1)	homozygous	SV, 46, XY + hyperpigmented scrotum, hypospadias
*Alos N. 2000 (* 32 *)*	c.29C>A	p.Ala10Glu	French-Canadian (2)	homozygous	SW (2/2); 46,XY + ambiguous genitalia, azoospermic, TART ; 46,XX + progressive breast development, menarche at 10 y.o., enlarged ovaries with cysts
*Moisan AM. 1999 (* 36 *)*	c.29C>T	p.Ala10Val	Egyptian (2)	homozygous	SV 46, XY + hypospadias (2/2), siblings
*Benkert AR. 2015 (* 42 *)*	c.35G > A	p.Gly12Glu	American/Amish (16)	homozygous	46, XY (6/16), 46, XX (10/16), SW (6/16), TART (2/16), hypospadias (6/16), PCOS (2/16), hirsutism/acne (5/16)
*Rhéaume E. 1995 (* 52 *)*	c.44G>A	p.Gly15Asp	Algerian (1)	homozygous	SW 46, XY + hypospadias
*Øzdemir CM. 2024 (* 43 *)*	c.65T>C	p.Leu22Ser	Danish (2)	heterozygous	SV 46,XX + growth acceleration, hirsutism, primary amenorrhoea, PCOS, acne, diagnosed at 23 y.o., SV 46,XX + dysmenorrhoea, hirsutism, diagnosed at 25 y.o.
*Huang Y. 2014 (* 41 *)*	c.73G>T	p.Glu25*	Chinese (1)	homozygous	SW 46XX + clitoromegaly and recurrent ovarian cysts
*Fanis P. 2020 (* 53 *)*	c.106A>T	p.Lys36*	Roma/ Cypriot (1)	homozygous	SW 46,XY + ambigous genitalia, gynecomastia, adrenarche at 3,5 y.o. + p.Val281Leu in the CYP21A2 gene
*Dundar A. 2019 (* 54 *)*	c.142+1 G>T	p.?	nd	homozygous	nd
*Morel Y. 2014 (* 55 *)*	c.142+2T>C	p.?	Polish (1)	nd	nd
*Chang HA. 2023 (* 56 *)*	c.-149_143-1766del	p.?	Korean (1)	heterozygous	SW 46,XY + hyperpigmentation
*Chen L. 2021 (* 13 *)*	c.154_162delinsTCCTGTT	p.Arg52Serfs*7	Chinese (1)	heterozygous	SW 46, XY + hypospadias, micropenis, intellectual and developmental retardation
*Lolis E. 2018 (* 45 *)*	c.214T>C	p.Cys72Arg	Swedish (1)	homozygous	SW 46,XY + hypospadias, micropenis, cryptorchidism, bifid scrotum, advanced bone age, TART, infertility
*Marui S. 2000 (* 40 *)*	c.222C>A	p.Asp74Glu	Brazilian (2)	heterozygous	SV 46,XX (2/2), premature pubarche at 6-7 y.o (1/2)
*Codner E. 2004 (* 57 *)*	c.232G>A	p.Val78Ile	Chilian (1 control)	heterozygous*	healthy carrier
*Moisan AM. 1999 (* 36 *) , Mendonca BB. 1994 (* 39 *), Teasdale SL. 2017 (* 58 *)*	c.244G>A	p.Ala82Thr	Brazilian (4), British (1)	homozygous (4/5), heterozygous (1/5)	Family 1: NC, 46, XX - clinically normal , SW 46,XY + ambigous genitalia, assigned at birth as female (2/3), 46, XY- female phenotype in adulthood (1/3), 46, XY - male phenotype in adulthood (1/3); 2 family: NC, 46, XX - premature pubarche at 5y.o.; Family 3: SV 46,XY + female phenotype, ambigous genitalia
*Rabbani B. 2012 (* 59 *)*	c.244G>C	p.Ala82Pro	Iranian (1)	homozygous	SW, 46, XY + hyperpigmentation, microphallus, hypospadias, inguinal hernia
*Nordenstrom A. 2007 (* 34 *)*	c.245C>A	p.Ala82Asp	Russian (1)	heterozygous	SW; 46XX + premature pubarche 3 months old
*Guran T. 2020 (* 49 *)*	0.274_275delCA	p.H92Qfs*32	Turkish (1)	homozygous	SW, 46,XX +DSD
*Mebarki F. 1995 (* 60 *), McCartin S. 2000 (* 61 *)*	c.299A>G	p.Asn100Ser	Algerian (1), English (2)	homozygous (1/3), heterozygous (2/3)	Family 1: SV, 46, XY + ambiguous genitalia (1/3); Family 2: SV, 46,XY + premature adrenarche, hypospadias, micropenis (1/3); SW, 46, XY + cutaneous hyperpigmentation (1/3)
*Limatta J. (2025) (* 62 *)*	c.307+1G>A	p.?	African/South American (1)	heterozygous	SV 46,XY + ambiguous genitalia, female sex registration at birth and female identity, autism spectrum disorder, premature adrenarche and pubarche at 5 y.o., bone age advancement
*Present study, Menegatti E. 2022 (* 6 *)*	**c.308-6G>A**	**p.?**	**Polish (1),** Italian (2)	**heterozygous**	**Family 1 (Present study): SV, 46,XY + hypospadias, hyperpigmented bifid scrotum, micropenis;** Family 2: SW 46, XY + hypospadias, hyperpigmented bifid scrotum, SV, 46,XY + hypospadias
*Guran T. 2020 (* 49 *)*	c.320T>A	p.Leu107Gln	Turkish (1)	homozygous	SW, 46,XY +DSD
*Morel Y. 2014 (* 55 *), Moisan AM. 1999 (* 36 *)*,	c.323T>G	p.Leu108Trp	Spanish/Portuguese (1)	heterozygous	SW 46,XY + hypospadias, bifid scrotum
*Morel Y. 2014 (* 55 *)*	c.367A>G	p.Ser123Gly	French (1)	nd	nd
*Menegatti E. 2022 (* 6 *)*	c.370A>G	p.Ser124Gly	Italian (2)	heterozygous	SW 46, XY + hypospadias, hyperpigmented bifid scrotum, SV, 46,XY + hypospadias
*Morel Y. 2014 (* 55 *)*	c.370_372del	p.Ser124del	Pakistani (1)	nd	nd
*Hathi D. (2022) (* 63 *)*	c.371G>T	p.Ser124Ile	Indian (1)	homozygous	SW 46,XY + ambiguous genitalia, hyperpigmentation
*Dundar A. 2019 (* 54 *)*	c.380 T>A	p.Val127Glu	Turkish (1)	homozygous	nd
*Rhéaume E. 1994 (* 38 *), Chang YT. 1993 (* 64 *), Pang S. 1983 (* 65 *), Marui S. 2000 (* 40 *)*	c.385G>A	p.Gly129Arg	American (4), Brazilian (2)	heterozygous	Family 1: SV, 46, XY + cryptorchidism, hypospadias, premature pubarche, growth acceleration at 6 y.o., SV 46, XX + premature pubarche at age 4y.o, clitoromegaly; Family 2: SV 46, XX + irregular menses, hirsutism, enlarged ovaries with multiple microcysts, SV, 46, XY + cryptorchidism, hypospadias; Family 3: SV 46,XX (2/2), premature pubarche at 6-7 y.o (1/2)
*Marui S. 1998 (* 66 *)*	c.403G>T	p.Glu135*	Chilian (1)	homozygous	SW 46, XX + hyperpigmented external genitalia
*Cara JF. 1985 (* 67 *), Simard J. 1993 (* 68 *), Pang S. 2002 (* 33 *), Chen L. 2021 (* 13 *), Panzer K. 2017 (* 69 *)*	c.424G>A	p.Glu142Lys	American (3), Chinese (1)	heterozygous (3/4), homozygous (1/4)	Family 1: SW 46, XY + hypospadias, bifid scrotum; Family 2: SV 46, XX + premature pubarche (5,5y), delayed bone age, acne; Family 3: SW 46,XY + micropenis, hypospadias, Family 4: SW 46,XY + dysmorphic facial features (frontal bossing, hypotelorism, low nasal bridge, anteverted nares), hypospadias, bifid scrotum
*Guran T. 2020 (* 49 *)*	c0.429_430insAA	p.E144Kfs*31	Turkish (2)	homozygous	Family 1: SW 46, XY +DSD, SW 46,XX
*Ladjouze A.2022 (* 24 *)*	c.453_464del	p.Thr152_Pro155del	Algerian (2)	homozygous	Family 1: SW 46,XX (2/2), DSD (1/2)
*Moisan AM. 1999 (* 36 *)*,	c.464C>T	p.Pro155Leu	French (2)	heterozygous	Family 1: SV 46, XY + hypospadias (2/2)
*Dung VC. 2015 (* 70 *)*	c.481G>C	p.Ala161Pro	Vietnamese (2)	homozygous	Family 1: SW 46,XY + ambiguous genitalia; Family 2: SW 46,XY + ambiguous genitalia
*Probst-Scheidegger U. 2016 (* 71 *)*	c.503delC	p.Ala168Valfs*6	Swiss (1)	heterozygous	SW 46, XX
*Almaramhy HH. 2023 (* 72 *)*	c.507T>A	p.Asn169Lys	Yemeni (2)	homozygous	Family 1: 46,XY + hypospadias (2/2)
*Cara JF. 1985 (* 67 *), Simard J. 1993 (* 68 *), Rhéaume E. 1992 (* 73 *), Morel Y. 2014 (* 55 *), Probst-Scheidegger U. 2016 (* 71 *)*	c.512G>A	p.Trp171*	American (2), Swiss (3), South Indian (1)	heterozygous (3/6), homozygous (2/6), nd (1/6)	Family 1: SW 46, XY + hypospadias, bifid scrotum; Family 2: SW 46, XY + hypospadias, gynecomastia, male sibling died as a newborn propably due to adrenal crisis; Family 3: SW 46,XX + delayed puberty, male sibling died as a newborn propably due to adrenal crisis ; Family 4: SW 46,XX + delayed puberty, Family 5: nd, Family 6: SW 46, XX
*Moisan AM. 1999 (* 36 *), Russel AJ. 1994 (* 74 *), Alkhatib EH. 2021 (* 75 *)*,	c.518T>G	p.Leu173Arg	Scottish (1), American (1)	heterozygous	Family 1: SV 46,XY + hypospadias; Family 2: SW 46, XY +hypospadias
*Wiromrat P. 2015 (* 76 *)*	c.540C>A	p.Tyr180*	Thai/Indian (1)	homozygous	SW 46,XY + ambiguous genitalia
*Johannsen TH. 2005 (* 37 *)*	c.542C>T	p.Thr181Ile	Danish (2)	heterozygous	Family 1: SW 46,XX (2/2), premature pubarche (7y7m), slight growth acceleration, and advanced bone age (1/2)
*Morel Y. 2014 (* 55 *)*	c.555A>C	p.Arg185Ser	French (1)	nd	SW
*Moisan AM. 1999 (* 36 *), Morel Y. 2014 (* 55 *)*	c.557C>T	p.Pro186Leu	Spanish/Portugese (1)	heterozygous	SW 46,XY + hypospadias, bifid scrotum
*Rhéaume E. 1992 (* 73 *), Øzdemir CM. 2024 (* 43 *)*	c.558dup	p.Thr187Hisfs*17	American (1), Dutch (1), Danish (2)	heterozygous	Family 1: SW 46, XY + hypospadias, gynecomastia, male sibling died as a newborn propably due to adrenal crisis; Family 2: SW; Family 3: SV 46,XX + growth acceleration, hirsutism, primary amenorrhoea, PCOS, acne, diagnosed at 23 y.o., SV 46,XX + dysmenorrhoea, hirsutism, diagnosed at 25 y.o.
*Takasawa K. 2014 (* 77 *)*	c.569A>G	p.Tyr190Cys	Japanese (1)	heterozygous	SV 46,XX + labia minora fusion, clitoromegaly
*Yadav BR. 2022 (* 78 *)*	c.590T>C	p.Leu197Pro	Indian (1)	homozygous	SV 46,XY + hypospadias, micropenis, bifid scrotum with hyperpigmentation
*Katsumata N. 1995 (* 79 *)*	c.614T>C	p.Leu205Pro	Japanese (2)	homozygous	Family 1: SW 46, XY + hypospadias, bifid scrotum, SW 46,XX + clitoromegaly, hyperpigmentation
*Codner E. 2004 (* 57 *)*	c.638G>C	p.Ser213Thr	Chilean (1)	heterozygous	46, XY + hypospadias, cryptorchidism, precocious pubarche
*Moisan AM. 1999 (* 36 *)*	c.637A>G	p.Ser213Gly	American (1)	nd	SV 46, XX + Premature pubarche at 4 y, growth acceleration
*Morel Y. 2014 (* 55 *), Guran T. 2020 (* 49 *), Dundar A. 2019 (* 54 *), Takasawa K. 2014 (* 77 *)*	c.652T>C	p.Ser218Pro	Turkish (6), Japanese (1)	heterozygous (1/6), nd ( 5/6)	SW (5/5), 46,XY+DSD (3/5), 46,XX (1/5); SV 46,XX + labia minora fusion, clitoromegaly
*Pang S. 2002 (* 33 *), Levy-Shraga Y. 2016 (* 80 *)*	c.664C>A	p.Pro222Thr	American/Eastern European Jewish (1), Jewish (1)	homozygous	SW 46,XX (2/2)
*Morel Y. 2014 (* 55 *), Moisan AM. 1999 (* 36 *), Lusa LG. 2010 (* 81 *), Ladjouze A. 2022 (* 24 *), Araújo VG. 2014 (* 82 *)*	c.665C>A	p.Pro222Gln	Algerian (14), Brazilian (2), French Arab (2), Colombian (3)	homozygous (16/21), nd (5/21)	Family 1: SW 46,XY + hypospadias, micropenis, SW 46,XX + clitoromegaly; Family 2: SW 46,XY + ambiguos genitalia; 12/20 SW, 8/20 DSD, Family 3: SW 46,XY + ambiguos genitalia ; nd (5/20)
*Li Z. 2021 (* 46 *), Yu L. 2021 (* 47 *)*	c.674 T > A	p.V225D	Chinese (4)	heterozygous	Family 1: 46,XY + premature pubarche (20ml testes at 9 y.o), dark skin, accelerated growth; Family 2: 46, XY + TART surgically removed, 46, XY + TART surgically removed; Family 3: SW 46,XY + micropenis, hypospadias
*Donadille B. 2018 (* 25 *)*	c.687del	p.Trp230Glyfs*7	French (1)	homozygous	SW 46,XY + micropenis, hypospadias
*Moisan AM. 1999 (* 36 *), Burckhardt MA. 2015 (* 83 *)*	c.687_713del	p.Trp230_Ala238del	Sri Lanka (3)	homozygous	Family 1: SW 46,XY + hypospadias, micropenis; Family 2: SW 46,XY + hypospadias, Family 3: SW, 46XY + hypospadias, cryptorchidism and undervirilization
*Nordenstrom A. 2007 (* 34 *), Melikian MA. 2008 (* 84 *)*	c.690G>A or c.689G>A	p.Trp230*	Russian (3)	heterozygous (1/3), homozygous (2/3)	Family 1: SW; 46XX + premature pubarche 3 months old; Family 2: SW 46,XY + false male hermaphroditism, SW 46,XX + ambiguous genitalia, moderate virilization
*Nicola AG. 2022 (* 19 *), Claahsen- van der Grinten HL. 2008 (* 48 *), Alkhatib EH. 2021 (* 75 *)*	c.694C>G	p.His232Asp	Dutch (1), American (1)	homozygous (1/2), heterozygous (1/2)	Family 1: 46,XY + TART at 16 y.o., short stature (-3,5 SDS), adrenal rest tumor in perirenal region at 23 y.o; Family 2: SW 46,XY +hypospadias
*Moisan AM. 1999 (* 36 *)*	c.707T>C	p.Leu236Ser	French (1), American (2)	heterozygous(1/3), homozygous (2/3)	Family 1: SV 46, XY + hypospadias, micropenis; Family 2: 46,XX + premature pubic hair; Family 3: 46,XX + hirsutism, oligomenorrhea
*Simard J. 1993 (* 68 *), Heinrich UE. 1993 (* 85 *), Guran T. 2020 (* 49 *)*	c.733G>C	p.Ala245Pro	Turkish (3)	homozygous	Family1: SV 46, XY + hypospadias,bifid scrotum, 4 of 7 siblings died in early infancy from undetermined causes; Family 2: SW, 46,XY + DSD (2/3)
*Tajima T. 1995 (* 86 *), Giri D. 2020 (* 87 *), Yoshimoto M. 1997 (* 88 *), Chang HA. 2023 (* 56 *)*	c.745C>T	p.Arg249*	Japanese (3), British/ British-Afro-Caribbean (1), Korean (1)	homozygous (4/5), heterozygous (1/5)	Family 1: SW 46, XY + hypospadias, bifid scrotum; Family 2: SW 46, XY + clitoromegaly; Family 3: SW 46,XX + Bartter syndrome type 3, Family 4: SW 46, XY + hypospadias, bifid scrotum, micropenis, gynecomastia at 7,5 y.o., Family 5: SW 46,XY + hyperpigmentation
*Baquedano MS. 2015 (* 12 *)*	c.749G>T	p.Gly250Val	Argentinian (1)	homozygous	SV 46,XX + clitoromegaly, advanced bone age, precocious pubarche (PII,PIII) at 7 m.o.
*Simard J. 1993 (* 68 *), Morel Y. 2014 (* 55 *)*	c.757T>A	p.Tyr253Asn	Dutch (1)	heterozygous	SW
*Rosenfield RL. 1980 (* 30 *), Sanchez R. 1994 (* 29 *); Present study*	**c.760T>G**	**p.Tyr254Asp**	**Polish (3)**	**heterozygous (2/3), homozygous (1/3)**	Family 1: SV 46XX + primary amenorrhea, hirsutism, acne**; Family 2 (Present study): SW 46,XX + cushingoid ; Family 3(Present study): SV, 46,XY + hypospadias, hyperpigmented bifid scrotum, micropenis**
*Tajima T. 1995 (* 86 *)*	c.776C>G	p.Thr259Arg	Japanese (2)	homozygous	Family 1: SW 46,XY + hypospadias, bifid scrotum, SW 46,XX
*Zhang L. 2000 (* 51 *), Moisan AM. 1999 (* 36 *), Li Z. 2021 (* 46 *), Yu L. 2021 (* 47 *), Chen L. 2021 (* 13 *), Leka-Emiri S. 2022 (* 89 *), Wiromrat P. 2015 (* 76 *)*	c.776C>T	p.Thr259Met	Taiwanese (1), French (2), Brazilian (2), Chinese (4), Afghan (1), Thai/Indian (1)	homozygous (4/11) heterozygous (7/11)	Family 1: SW; 46XY + with female phenotype, ambiguous genitalia, micropenis, hypospadias; Family 2: SW 46, XY + hypospadias, bifid scrotum, SW 46, XY + normal genitalia; Family 3: SV 46,XX + clitoromegaly, severe virilization; Family 4: SV 46,XX + clitoromegaly; Family 5: 46,XY + premature pubarche (20ml testes at 9 y.o), dark skin, accelerated growth; Family 6: 46, XY + TART surgically removed, 46, XY + TART surgically removed; Family 7: SW, 46,XX +clitoromegaly; Family 8: SW 46,XY + micropenis, hypospadias; Family 9: SW 46,XY +micropenis, hypospadias
*Liimatta J. 2025 (* 62 *)*	c.779C>T	p.Pro260Leu	African/South American (1)	heterozygous	SV 46,XY + ambiguous genitalia, female sex registration at birth and female identity, autism spectrum disorder, premature adrenarche and pubarche at 5 y.o., bone age advancement
*McCartin S. 2000 (* 61 *)*	c.797delA	p.Asn266Thrfs*6	English (2)	heterozygous	SV, 46,XY + premature adrenarche, hypospadias, micropenis (1/3); SW, 46, XY + cutaneous hyperpigmentation (1/3)
*Simard J. 1994 (* 90 *), Zhang L. 1996 (* 91 *), Leka-Emiri S. 2022 (* 89 *)*	c.818_819del	p.Lys273Argfs*7	Afghan/Pakistani (4) Afghan (1)	homozygous(3/5), heterozygous (2/5)	Family 1: SW 46, XY + hypospadias, ambigous genitalia; Family 2: SW 46, XY + hypospadias, bifid scrotum, ambigous genitalia; Family 3: SW 46, XY + hypospadias, bifid scrotum, ambigous genitalia, male infant brother died suddenly at 37 days of age; Family 4: SW 46, XX + clitoromegaly, public hair growth during infancy, two siblings and two cousins with ambiguous genitali died during early infancy with adrenal crisis symtoms; Family 5: SW 46,XY + micropenis, hypospadias
*Present study*	**c.849delG**	p.Trp283* / frameshift mutation	**Polish (1)**	**homozygous**	**SW 46, XX + premature pubarche at 7y3m, clitoromegaly, ovarian cysts**
*Codner E. 2004 (* 57 *), Morel Y. 2014 (* 55 *)*	c.852C>G	p.Ser284Arg	Chilian (1)	heterozygous	SV 46,XY + hypospadias
*Moisan AM. 1999 (* 36 *)*	c.867delG	p.Met290Cysfs*10	French (2)	heterozygous	Family 1: SW 46, XY + hypospadias, bifid scrotum, SW 46, XY + normal genitalia
*Moisan AM. 1999 (* 36 *)*	c.881G>T	p.Gly294Val	French (2)	heterozygous	Family 1: SV 46, XY + hypospadias (2/2)
*Shehab MA 2018 (* 35 *)*	c.895G>A	p.Val299Ile	Bangladeshi (1)	homozygous	47,XXY/46,XX + microphallus, hypospadias, cryptorchidism, advanced bone age, premature pubarche, hyperpigmentation + 2 other pathogenic variants in HSD3B2 gene
*Guran T. 2020 (* 49 *)*	c.911T>C	p.Leu304Pro	Turkish (2)	homozygous	SW 46,XY + DSD
*Tajima T. 1995 (* 86 *)*	c.924C>G	p.Tyr308*	Japanese (1)	nd	SW 46,XY + hypospadias, bifid scrotum, brother died as neonate due to salt-losing crisis
*Shehab MA. 2018 (* 35 *)*	c.925T>A	p.Ser309Thr	Bangladeshi (1)	homozygous	47,XXY/46,XX + microphallus, hypospadias, cryptorchidism, advanced bone age, premature pubarche, hyperpigmentation + 2 other pathogenic variants in HSD3B2 gene
*Morel Y. 2014 (* 55 *), Teasdale SL. 2017 (* 58 *)*	c.931C>T	p.Gln311*	French/Caucasian (1), British (1)	heterozygous (1/2) nd(1/2)	SWs (1/2), SV 46,XY + female phenotype, ambigous genitalia (1/2)
*Shehab MA. 2018 (* 35 *)*	c.932A>G	p.Gln311Arg	Bangladeshi (1)	heterozygous	47,XXY/46,XX + microphallus, hypospadias, cryptorchidism, advanced bone age, premature pubarche, hyperpigmentation + 2 other pathogenic variants in HSD3B2 gene
*Guran T. 2020 (* 49 *)*	c0.934delC	p.F314Sfs*54	Turkish (1)	homozygous	SW 46,XY + DSD
*Zhang L. 1996 (* 91 *)*	c.953delC	p.Thr318Lysfs*50	Pakistani (1)	heterozygous	SW 46, XX + clitoromegaly, public hair growth during infancy, two siblings and two cousins with ambiguous genitali died during early infancy with adrenal crisis symtoms
*Bizzarri C. 2016 (* 92 *)*	c.956del	p.Val319Alafs*49	Italian/Sardinian (1)	homozygous	SW 46,XY + hypospadias, micropenis
*Guran T. 2020 (* 49 *), Ertorer ME. 2024 (* 93 *)*	c.959_960insC	p.Leu321Ilefs*4	Turkish (2)	homozygous	SW 46,XY + DSD (2/2)
*Guran T. 2020 (* 49 *), Dundar A. 2019 (* 54 *)*	c.967A>G	p.Asn323Asp	Turkish (14)	homozygous	SW 46,XY + DSD (7/14), SW 46,XX (7/14)
*Scaramuzzo RT. 2017 (* 94 *), Mellone S. 2022 (* 95 *)*	c.969T>G	p.Asn323Lys	Moroccan (4)	homozygous	Family 1: SW 46,XX (2/2); Family 2: twins - SW 46, XX, SW 46,XY + hypospadias, small penis, hyperpigmented and fused scrotal folds
*Jeandron DD. 2012 (* 96 *)*	c.1000C>T	p.Gln334*	American/Salvadoran (1)	homozygous	SW 46,XX + increased pigmentation of the areolae and labia
*Welzel M. 2008 (* 97 *), Chen L. 2021 (* 13 *), Dundar A. 2019 (* 54 *)*	c.1003C>T	p.Arg335*	Turkish (3), Chinese (2)	homozygous (2/5), heterozygous (3/5)	Family 1: SW 46,XY + hypospadias, micropenis, broad urogenital sinus, cryptorchidism, urethroplasty, SW 46,XY + hypospadias, micropenis, cryptorchidism, incomplete cleft lip, urethroplasty; Family 2: SW 46, XY + hypospadias, micropenis, intellectual and developmental retardation; Family 3: SW, 46,XX +clitoromegaly
*Welzel M. 2008 (* 97 *)*	c.1022C>T	p.Pro341Leu	Lebanese (1)	homozygous	SW 46,XY + hypospadias, micropenis, broad urogenital sinus
*Guran T. 2020 (* 49 *), Güven A. 2017 (* 50 *)*	c.1063T>C	p.Trp355Arg	Turkish (2)	homozygous	Family 1: SW 46,XY + hypospadias, left cryptorchidism, bifid scrotum, TART at 3 y.o., SW 46,XY + hypospadias, TART at 2 y.o.
*Welzel M. 2008 (* 97 *)*	c.1064G>A	p.Trp355*	Bangladeshi (1)	homozygous	SW 46,XY + hypospadias, small penis
*Guran T. 2020 (* 49 *)*	c.1076T>C	p.Leu359Pro	Turkish (2)	homozygous	SW 46, XY, SW 46,XX
*Pan Y. 2012 (* 98 *)*	c.1088C>T **	–	Chinese (2)	homozygous	Family 1: SW 46,XY SW 46,XY, hyperpigmentation (2/2)
*Johannsen TH. 2005 (* 37 *)*	c.1103delA	p.Lys368Serfs*72	Danish (2)	heterozygous	Family 1: SW 46,XX (2/2), premature pubarche (7y7m), slight growth acceleration, and advanced bone age (1/2)
*Pang S. 2002 (* 33 *)*	c.1119A>C	p.*373Cysext*95	American (1)	heterozygous	SV 46, XX + premature pubarche (5,5y), delayed bone age, acne
*Pan Y. 2012 (* 98 *)*	c.1132C>G ***	–	Chinese (2)	homozygous	Family 1: SW 46,XY, SW 46,XY, hyperpigmentation (2/2)

*Single pathogenic variant

**Wrong ref. Ref at this location is A (31)

***Illegal start position in cdot (31)

p.?, variant causes major aminoacid change; SW , salt wasting; SV’, simple virilizing; TART, testicular adrenal rest tumor; PCOS, polycystic ovary syndrome; DSD, differences in sex development

Mutations of cases presented in this article are marked in bold.

This study presents three pediatric cases of 3β-HSD2D diagnosed at the Pediatric Endocrinology Department, University Children’s Hospital in Krakow, highlighting their clinical features, genetic findings, and management strategies.

## 2 Materials and methods

### 2.1 Retrospective analysis of patients’ medical files

**Figure 2:**
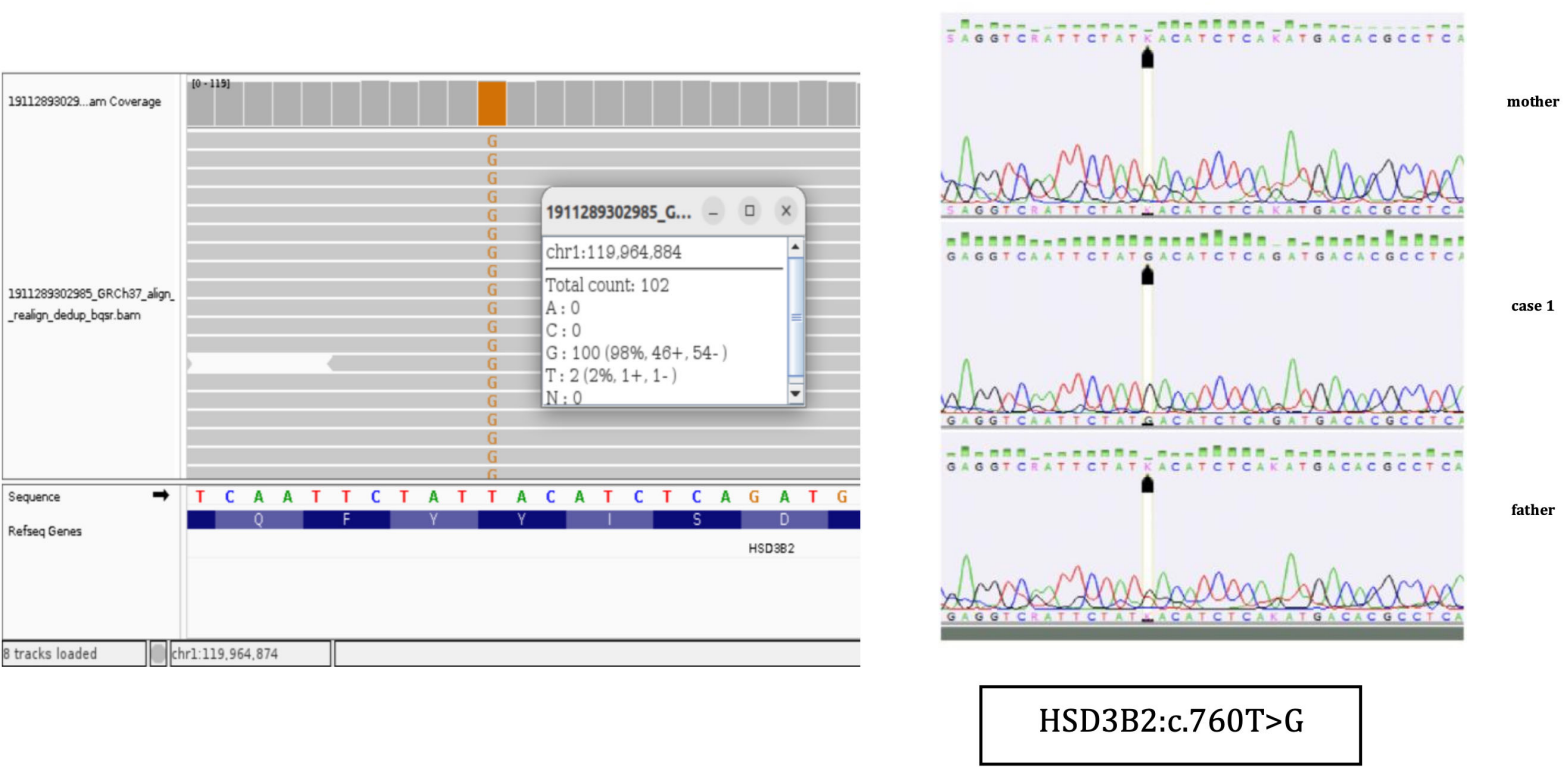
NGS results and Sanger sequencing chromatograms of the homozygous NM000198.4 (*HSD3B2*):c.760T>G (p.Tyr254Asp) variant in Case 1.

**Figure 3:**
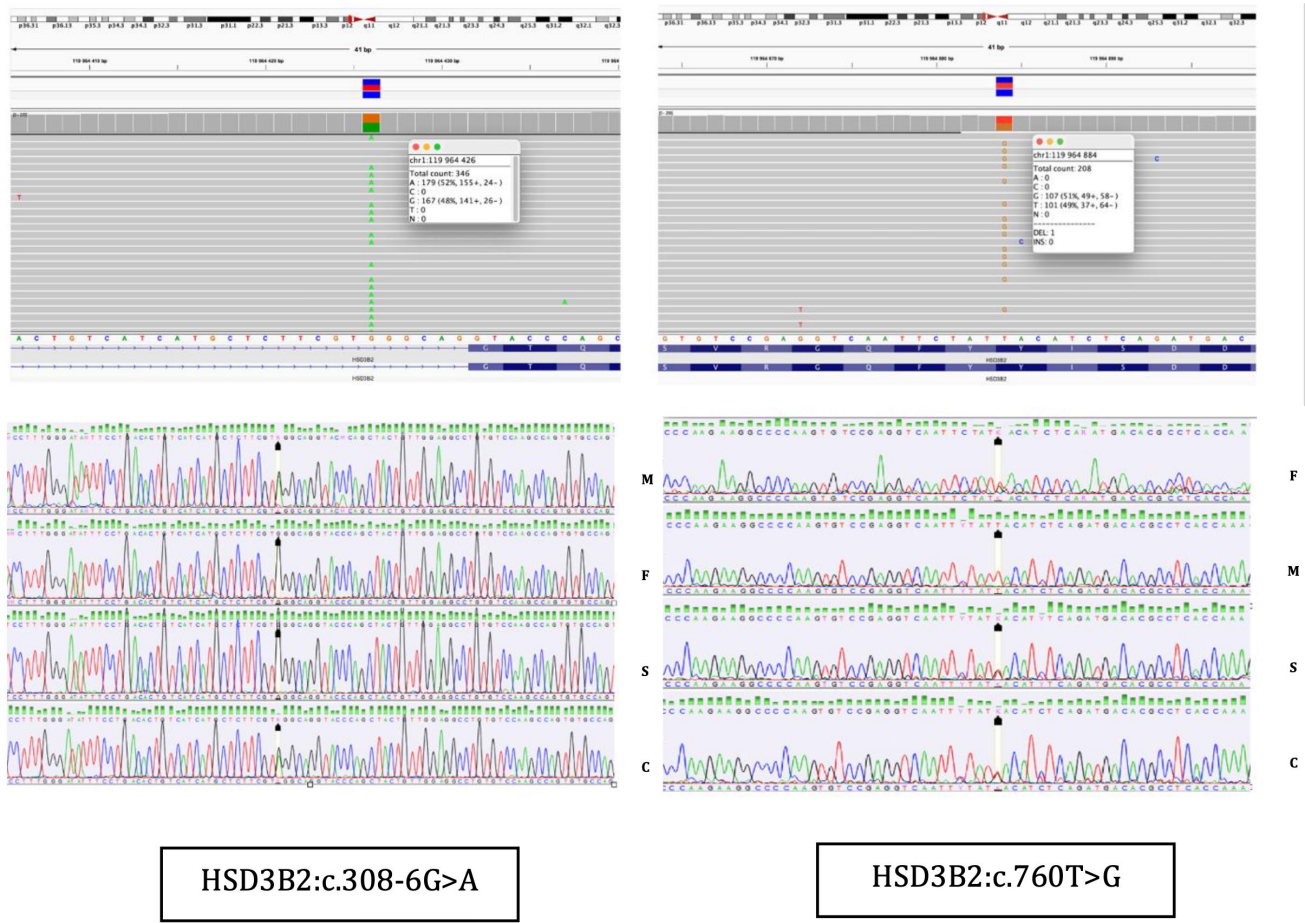
NGS results and Sanger sequencing chromatograms of the heterozygous NM000198.4 (*HSD3B2*):c.760T>G (p.Tyr254Asp) and NM_000198.4(*HSD3B2*): c.308-6G>A variants in Case 2. F, father; M, mother; S, sister; C, Case 2.

**Figure 4:**
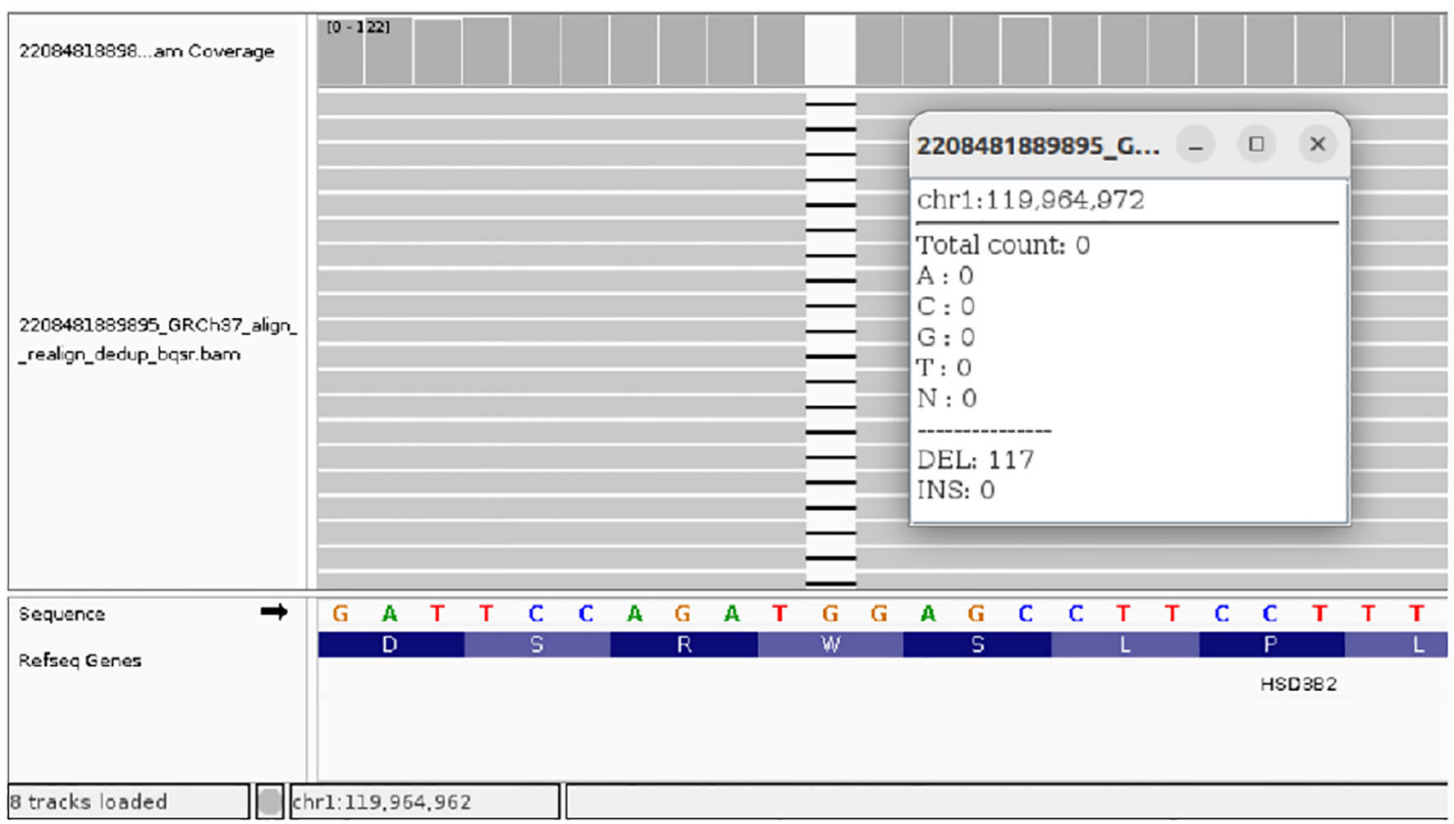
NGS results of the homozygous NM_000198.4(*HSD3B2*): c.849delG (p.Trp283fs) variant in Case 3.

**Figure 5:**
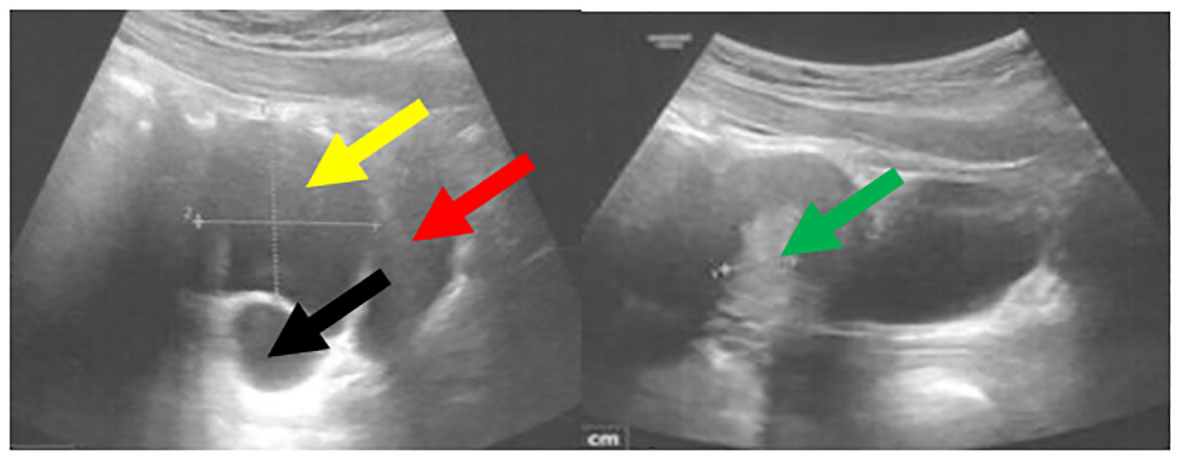
Pelvic ultrasound performed at 13 years and 8 months of age demonstrated a cyst within the right ovary measuring 5 × 4 cm. The bladder is indicated by the red arrow, the uterus with an endometrial thickness of 13.3 mm by the green arrow, the cyst by the yellow arrow, and the right ovary by the black arrow.

**Figure 6:**
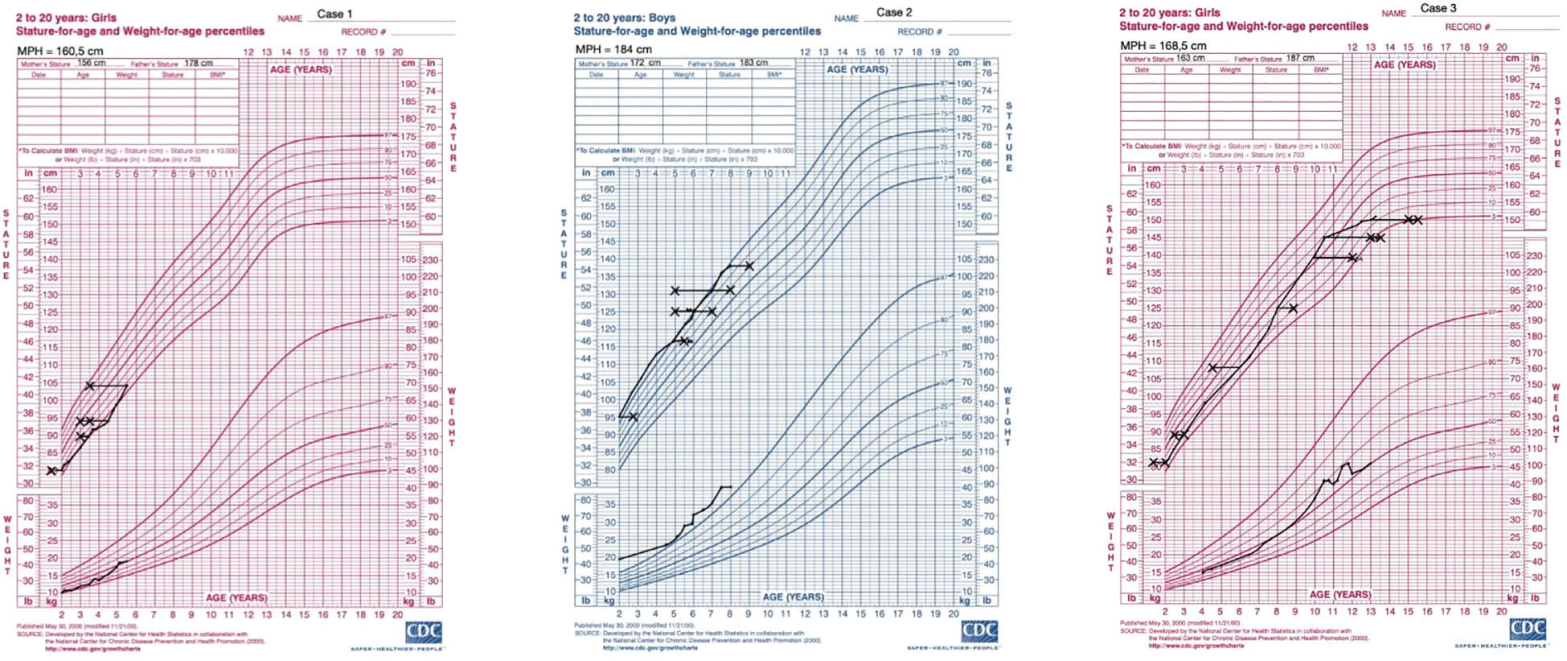
Percentile growth charts of weight and height for the presented cases. The charts were obtained from the CDC website (https://www.cdc.gov/growthcharts/cdc-charts.htm). Legend: X- marks bone age.

**Figure 7:**
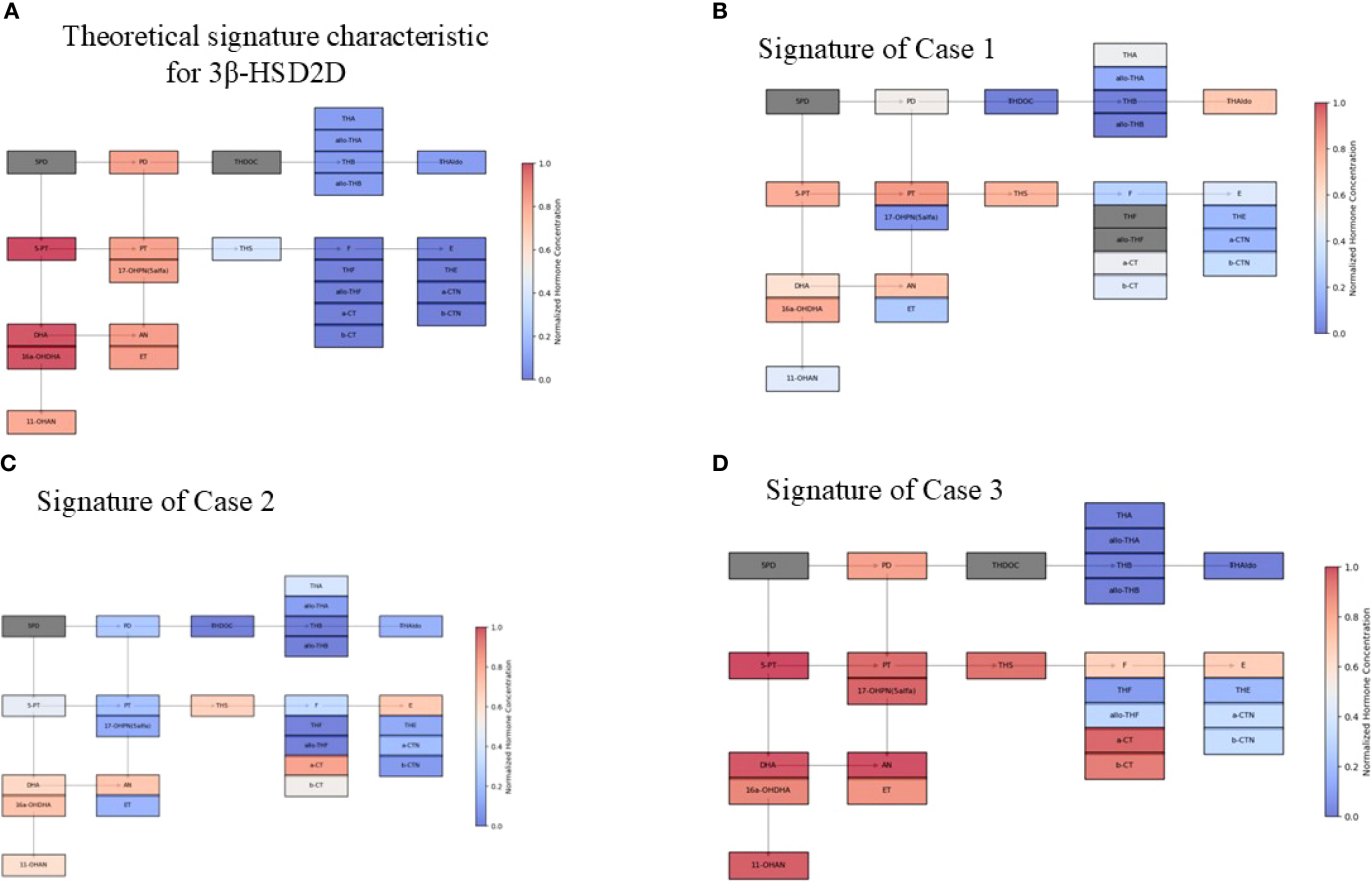
Schematic visualization of urine steroid profile signature Concentrations were normalized using the upper limit of the norm, such that highly increased values approach 1.0 and decreased concentrations tend to 0.0. Hormones not tested are greyed out. **(A)** Theoretical signature characteristic for 3β-HSD2D. **(B)** Signature of Case 1 **(C)** Signature of Case 2 **(D)** Signature of Case 3 *Abbreviations of urine steroid metabolites (in brackets the corresponding general precursors in serum): 5PD*, *5-Pregnanediol (Pregnenolone); 5-PT, 5-Pregnenetriol (17α-hydroxypregnenolone); PD*, *Pregnanediol (Progesterone); PT, Pregnanetriol (17α-hydroxyprogesterone); 17-OHPN(5 α)*, *5- α 17-OH-Pregnanolone; DHA, Dehydroepiandrosterone; 16a-OHDHA*, *16 α -OH-Dehydroepiandrosterone; 11-OHAN, 11-OH- Androsterone (11 hydroxyandrostendione); AN, Androsterone (Androstenedione, Testosterone, 5 α-dihydrosterone); ET, Etiocholanolone (Androstenedione, Testosterone); THDOC, Tetrahydro-11-deoxycorticosterone (11-deoxycorticosterone); THA*, *Tetrahydro-11-dehydrocorticosterone (Corticosterone); AlloTHA*, *Allo-tetrahydro-11-dehydrocorticosterone (Corticosterone); THB, Tetrahydro-corticosterone (Corticosterone); Allo-THB, Allo-tetrahydro-corticosterone (Corticosterone); THAldo, Tetrahydro-aldosterone (Aldosterone); THS, Tetrahydro-11-deoxycortisol (11-deoxycortisol); F, Free cortisol; THF*, *Tetrahydro-cortisol; Allo-THF, Allo-tetrahydro-cortisol; a-CT, Alpha-ortolone; b-CT*, *Beta-cortolone; E*, *Cortisone; THE, Tetrahydro-cortisone*.

Retrospective analysis of patients’ medical files was performed (**Tables 2**–**4**, **Figures 2**–**7** **).** The patients were recruited between 2010 and 2025. During this period, we diagnosed a total of 135 individuals from 120 families with congenital adrenal hyperplasia (CAH), including 130 patients (96.3%) from 115 families with CAH due to 21-hydroxylase deficiency, 3 patients (2.2%) from 3 unrelated families with 3β-hydroxysteroid dehydrogenase type 2 deficiency (3βHSD2D), 1 patient (0.74%) with 11β-hydroxylase deficiency, and 1 patient (0.74%) with P450scc deficiency.

**Table 2:** Summary of the therapeutic management and longitudinal hormonal profile for Case 1, presenting key therapeutic and clinical data from birth to the age of 5 and a half years.

Age	HC [mg]	FC [mcg]	BP [mmHg]	B.a. GP	ACTH pg/ml [10–60]	Cort ng/ml [50–230]	17OHP ng/ml [0.03-0.82]	TST ng/ml [<1] androstendion (A) ng/ml [0.3-3.3]	Estradiol (E2) pg/ml [<7]; FSH mIU/ml [<3.3] LH mIU/ml [<5.5]	DHEA-S μg/dL	PRA ng/ml/h [1.5-5.7]	Aldosterone pg/ml [1–11 months 70-900, >11 months 35-310]	Electrolytes [mmol/l] [na-136-146 K-3.5-5.1 cl-101-109]	US adrenals/ ovaries
–							187.4 nmol/l 330.1 nmol/l [<35]							
9 days					1394	50	>20	A >10 ng/ml	–	–	–	–	Na-124 K-5.3 Cl-92	Right adrenal40x6.6 mm, left adrenal 40x9.6 mm
12 days (start hydrocortisone)	3 x 2.5 (39.5 mg/m2)	–	Reported as normal											
16 days (start fludrocortisone)	3 x 2.5 (39.5 mg/m2)	50	Reported as normal											
1 month	3 x 2.5	50			475.5	89	94.75	A >10 ng/ml	–	–	–	–	Na-131 K-5.7 Cl-96	Right adrenal 31x2.6 mm, left adrenal 35x4.5mm
2 months	3 x 2.5	50			573.5	39.2	7.9	T-0.27	E2 135.5; FSH 18.7; LH 5.5;	–	>30	46.8	Na 131 K-6.5 Cl-94.5	Right adrenal 13x8 mm, with hypoechogenic lesion 6 x 4 mm; left adrenal 14x7mm, with hypoechogenic lesion 5 x5 mm
2.5 m	5 + 2.5 + 2.5 (40 mg/m2)	2x50	Systolic 85-110											
3 m (cardiac surgery)	7.5 + 5+5 (70 mg/m2)	2x50	83/58		847.1	–	>15.3	–	–	–	>30		Na – 133 K – 6.28	Both adrenals not visible (normal).
6 months	3 x 2.5 (30 mg/m2)	2x50	68/42		4.4	50.7	0.08				<0.2	9.1	normal	–
7 months	3 x 2.5	2 x 50			–	–	–	T<0.1	E2-8.2; FSH-7.1; LH-0.24	10.2 [<85]	–	–	normal	–
9 m	3 x 2.5 (21.42 mg/m2)	2x50	88/62		5.4	30	0.12	–	–	–	<0.2	13.4	normal	–
1 y 2 m	3 x 2.5 (19.23 mg/m2)	2x50	121/85		5.0	179.1	0.13	–	–	–	<0.2	<7.6	normal	–
1 y 6 m	3 x 2.5 (17.4 mg/m2)	2x25	117/64		2.6	47.1	0.05	–	–	–	<0.2	<7.6	normal	–
1 y 9 m	2.5 + 2.5 + 2 (15.9 mg/m2)	2x25	98/68		7.0	40.2	<0.02	–	–	–	<0.2	<7.6	normal	–
2 y	2.5 + 2+2 mg (13.5 mg/m2)	2x25	–	1 y 6 m	48	45.8	0.08	–	–	3.3	0.94	16.2	K – 5.69	–
2 y 5 m					10.6	–	<0.02	T<0.1	–	–	<0.2	–	normal	
3 y	2.5 + 2+2 mg (12.5 mg/m2)	2x25	130/65		9.3	141.3	0.03	–	–	–	<0.2	–	normal	Right adrenal 15x5 mm, left adrenal not visible
3 y 6 m	2.5 + 2+2 mg (11.8 mg/m2)	2x25	98/60 86/61	3 y										
4 y 3 m	3 x 2.5 mg (12.29 mg/m2)	50 + 25	87/49		23.4	–	0.03	–	–	–	10.3	–	normal	Both adrenals not visible (normal). Uterus prepubertal 35x7x10 mm; ovaries 20x7x8 mm, with follicles up to 4 mm
4 y 7 m	3.75 + 2.5 + 2.5 (13.8 mg/m2)	50 + 25	96/64	3 - 3.5 y		–	<0.1	–	–	–	<0.2	–	–	–
5	3.75 + 2.5 + 2.5 (13.8 mg/m2)	50 + 25	103/71 93/66		25.3	Daily profile from <5 to max. 158.4	<0.1	T<0.1	–	<3.0	7.31	36.8	normal	Both adrenals not visible (normal)
5 y 6 m	3.75 + 2.5 + 2.5 (11.98 mg/m2)	50 + 25	93/60	3 y 6 m	21.3	–	0.10	–	–	–	11.3	–	normal	–

ACTH, Adrenocorticotropic Hormone; Cort., Cortisol; 17OHP, 17 Hydroxyprogesterone; TST (T), Testosterone; A, Androstendione; E2, Estradiol; FSH, Follicle Stimulating Hormone; LH, Luteinizing Hormone; DHEA S, Dehydroepiandrosterone Sulfate; PRA, Plasma Renin Activity; Aldos., Aldosterone; FIA, Fluoroimmunoassay; nmol/l, Nanomoles per liter; pg/ml, Picograms per milliliter; ng/ml, Nanograms per milliliter; mIU/ml, Milli international Units per milliliter; mcg/l, Micrograms per liter; ng/ml/h, Nanograms per milliliter per hour; mmol/l, Millimoles per liter; Na, Sodium; K, Potassium; Cl, Chloride; the mark> means the laboratory did not perform further dilution of the sample, and therefore, we do not have the exact value beyond this upper limit.

The table details the HC dosing regimens—provided both as absolute daily doses (in mg) and as calculated doses normalized to body surface area (mg/m²)—as well as fludrocortisone (FC) supplementation (in mcg). In addition, blood pressure (BP) measurements and bone age (B.A.) assessments using the Greulich-Pyle (GP) method are reported. Notable entries include the start of HC therapy, adjustments following cardiac surgery at 3 months, and variations in dosing and physiological parameters over time. Reference ranges for each hormone and electrolyte are indicated in brackets next to the respective parameter.

**Table 3:** Summary of the therapeutic management and longitudinal hormonal profile in Case 2 that outlines the chronological treatment approach, including the administration of intramuscular testosterone (TST) and daily hydrocortisone (HC) regimens.

Age	TST*	HC [mg]	BP [mmHg]	Bone age GP	Testes volume	ACTH pg/ml [10–60]	Cort ng/ml [50-230]	17OHP ng/ml [0.03-0.82]	Tst ng/ml [<1]	Estradiol (E2) pg/ml [<7]; FSH mIU/ml [<3.3] LH mIU/ml [<5.5]	DHEA-S μg/dL [NB-90-360; 1–5 y-<85; 6-11y <150; 12-17y 20-260]	PRA ng/ml/h [1.5-5.7]	Aldosterone pg/ml [1–11 months 70-900, >11 months 35-310]	Electrolytes [mmol/l] [na-136-146 K-3.5-5.1 cl-101-109]	US adrenals
35 weeks	20 mg im	–			2 ml										
36 weeks	20 mg im	–													
37 weeks	20 mg im	–													
6 days						547.4	200.7		1.38	FSH<0.3 LH<0.07					Enlarged R-length 40mm, L- length 34 mm
19 days						308.6	227.1			E2-274.1 FSH- 0.4 LH-2.3	>1500	8.38	246.8	Na-140 K-6.06 Cl-102.5	enlarged
2 months						95.1	103.3		0.83	E2-123.1 FSH- 0.6 LH-3.7	334.9			–	–
5 months						51.3	144.1	7.15			181.5	6.98		–	–
6 months								7.54			161.5	8.2		normal	–
8 months						53.3	88.4							normal	–
1 y 3 m	–			1 y 3 m-1 y 6 m		64.7	115	6.59			87	3.7		normal	–
1 y 8 m						60.1	97.8	4.39	<0.1		121.1	4.01		normal	–
1 y 10 m	–	Start ** 2x2.5 (7.3 mg/m2)			2ml										
2 y		2x2.5 (7.0 mg/m2)		2 y 8 m		35.1		3.44			137.9			normal	–
2 y 3 m		3x2.5 (10.5 mg/m2)				22.1		1.75			51.8			normal	–
3 y		2.5 + 3.75 + 2.5 (11.9 mg/m2)				9.1		1.35			39.9			normal	–
3 y 4 m								1.23				1.46		normal	–
4 y		2.5 + 3.75 + 2.5 (10.6 mg/m2)			2ml	14.5		1.89			60.4			normal	
4 y 7 m		2.5 + 5+2.5 (11.6 mg/m2)				5.9	165.4	1.35			86.1	4.72	152.5	K – 5.95	–
5 y		2.5 + 2.5 + 5 (11.6 mg/m2)	100/60	5 y 6 m	2ml			1.35				5.12		normal	–
5 y 7 m		5 + 3.75 + 2.5 (11.5 mg/m2)	103/68			6.2	68.6	0.89			73	5.9	60		
5 y 10 m						22.7	187.3					4.93		–	–
6		5 + 5+2.5 (12.37 mg/m2)	109/68	5 y- 7 y	2ml	8.6	306		<0.1	FSH- 0.8 LH-<0.07		1.82	35	–	–
6 y 10 m						10.1		1.36				3.58		normal	–
7 y		5 + 5+2.5 (11.16 mg/m2)	106/65	5 y- 8 y	3 ml										
7 y 2 m						9.8		2.31	<0.1		113.4	12.59		normal	–
8 y 4		5 + 5+5 (12.09 mg/m2)	102/69	9y	3ml	20.1						0.37		normal	–

*Testosterone enanthate was administered prior to hypospadias reconstruction surgery at 39 weeks of life.

**At 1 year 10 months, the HC regimen was introduced due to a rapid growth velocity of 15.9 cm/year and acceleration of bone age.

ACTH, Adrenocorticotropic Hormone; Cort., Cortisol; 17OHP, 17 Hydroxyprogesterone; TST, Testosterone; A, Androstendione; E2, Estradiol; FSH, Follicle Stimulating Hormone; LH, Luteinizing Hormone; DHEA S, Dehydroepiandrosterone Sulfate; PRA, Plasma Renin Activity; Aldos., Aldosterone; Na, Sodium; K, Potassium; Cl, Chloride; mIU, Milli international Units; mcg, Micrograms; mmol, Millimoles; l, Liter; m, Months; y, Years; NB, newborn; the mark> means the laboratory did not perform further dilution of the sample, and therefore, we do not have the exact value beyond this upper limit.

Dosages are presented both as absolute values (mg) and normalized to body surface area (mg/m²). The table also reports corresponding blood pressure (BP), bone age (B.A.) assessments using the Greulich-Pyle (GP) method, and testicular volume observations.

**Table 4:** Summary of the longitudinal treatment regimen and hormonal profile in Case 3, recorded from 4 months to 13 years and 3 months of age.

Age	Hc [mg]	FC [mcg]	BP mmHg	Bone age GP	Puberty	US adrenals/ ovaries	ACTH pg/ml [10-60]	Cort ng/ml [50-230]	17OHP ng/ml [0.03-0.82]	TST ng/ml [<1] androstendion (A) ng/ml [0.3-3.3]	Estradiol (E2) pg/ml [<7]; FSH mIU/ml [<3.3] LH mIU/ml [<5.5]	DHEA-S μg/dL	PRA ng/ml/h [1.5-5.7]	Aldosterone pg/ml [1–11 months 70-900, >11 months 35-310]	Electrolytes mmol/l [na-136-146 K-3.5-5.1 cl-101-109]
4 months (Before HC therapy)	5 + 5+2.5 (48 mg/m2)	50 + 50	90/60	–	THI A IPI	Right adrenal 17x8 mm, left adrenal 13x8 mm	1282	162.2	>9.5	T<0.1	FSH-3.46; LH-0.42	>822	5.61	510.9	Na-120 K-7.7 Cl-90
5 months (After HC Therapy)	5 + 2.5 + 2.5 (37 mg/m2)	50 + 50	–	–	THI A IPI	–	16.4					284.7			Na-139 K-5.2 Cl-103
5.5 m	5 + 2.5 + 2.5 (33.3 mg/m2)	50 + 25	–	–	THI A IPI	–			0.36				15.03		normal
6 m	5 + 5+2.5 (40.3 mg/m2)	50 + 25	–	–	THI A IPI	–									
7 m	5 + 5 + 2.5 (37.8 mg/m2)	50 + 25	–	–	THI A IPI	–			0.11				11.81		normal
8 m	5 + 3.75 + 2.5 (30.4 mg/m2)	50 + 25 + 25	–	–	THI A IPI	–									
11 m	5 + 2.5 + 2,5 (26.3 mg/m2)	25 + 25 + 25	110/60	–	THI A IPI	–							<0.2		normal
1y 2 m	5 + 2.5 + 2.5 (24.3 mg/m2)	2x25	–	–	THI A IPI	–									
1 y 6 m	5 + 5+2.5 (28.4 mg/m2)	2x25	–	1.5-3.5	THI A IPI	–									
2 y	5 + 5+2.5 mg (25.5 mg/m2)	2x25	–	1.5-2	THI A IPI	Adrenal glands not visible	4.6	242.7					<0.2		normal
2y 2 m	5 + 5+2.5 (24 mg/m2)	25	120/60		THI A IPI	Adrenal glands not visible									
3 y	5 + 2.5 + 2.5 (17.8 mg/m2)	25	90/60	2.5-3	THI A IPI	–									
3 y 6 m	5 + 2.5 + 2.5 (16.9 mg/m2)	25	–	–	THI A IPI	–	5.7					73	7.71		normal
4 y 2 m	3 x 2.5 (11.9 mg/m2)	25	105/60	–	THI A IPI	Uterus prepubertal ovaries normal Adrenal glands not visible	9.1					<3.0			normal
5	3x 2.5 (11.36 mg/m2)	25	100/60	–	THI A IPI	Adrenal glands not visible				T<0.1			>31.5		normal
6 y	3x2.5 (10 mg/m2)	50	–	4.5	THI A IPI	–	653.1						14.2		normal
7 y	3x2.5 (9.2mg/m2)	50	–	–	THI A IPI	–	713.1						10.36		normal
7 y 3 m	5 + 5+2.5 (14.7 mg/m2)	50 + 25	–	–	Th II PI	–									
8 y	5 + 5+2.5 (12.5 mg/m2)	50 + 25	110/60	8 y 10 m	Th II PI	Adrenal glands not visible	396.2					517.5	4.36		normal
8 y 5 m	5 + 5+2.5 (12.0 mg/m2)	50 + 25		–	Th III PI	–	349.7					471.2			normal
9 y	3x5 (13.6 mg/m2)	50 + 25	100/60	–	Th III PII	–						424.4	3.4		normal
9 y 5 m							63.7					173.3	8.34		normal
9 y 10 m							509.5		3.02		FSH-3.1 LH-4.97				normal
10 y	3x5 (12.6 mg/m2)	50 + 25	110/70	12 y	Th III PII	Adrenal glands not visible									
10 y 2 m							192.6		2.46	T-0.22	E2-49.5 FSH-11.9 LH-13.97		10.38		normal
10 y 7 m	6.25 + 5+5 (12.89 mg/m2)	50 + 37.5	120/70		Th IV PIII Menarche	–		21.9	7.51	T-0.4	E2-54.1 FSH-6.7 LH-5.38	520	6.52		normal
11 y	6.25 + 5+6.25 (13.77 mg/m2)	50 + 50	100/60	13-13.5 y	TH V PIV A(+) Regular menses	Endometrium 18 mm, 41x47x42 mm cyst in left ovary- Duphaston since 14 day of cycle; AFP <1.3; ng/ml B-HCG<2.0 mIU/m	472.1	87.5	5.92	T-0.21	E2-72.8 FSH-5.1 LH-6.98		4.38	25.9	normal
11 y 2 m							14.7		0.46	T-0.11	E2-61.4 FSH-2.6 LH-7.26		5.69		normal
11 y 6 m												284.4	15.54		
11 y 9 m	6.25 + 5+5 (11.86 mg/m2)	50 + 50	124/79	–	TH V PIV A(+) Regular menses	No cysts in ovaries on controlled US, Duphaston									
12 y	6.25 + 5+5 (12 mg/m2)	50 + 50	114/70	14-14.5 y	TH V PIV A(+) Regular menses	Adrenal glands not visible. Left ovary with follicles up to 10 mm; right ovary 57 x 28 x 29 mm with cyst 45x22x37 mm Duphaston; AFP <1,3 ng/ml; B-HCG <2,0 mIU/ml LDH 184,3 U/L	63		1.3				3.2		normal
12 y 2 m						Right ovary: 30 x 17 mm, with follicles up to 11; Left ovary 56 x 24 mm, with a anechogenic cyst 37 x 20 mm.	375.2			T – 0.25	E2 – 42.4 FSH – 6.5 LH - 5.23				
12 y 8 m	6.25 + 5+5 (12 mg/m2)	50 + 50	115/72	15 y	TH V PIV A(+) Regular menses	Adrenal glands not visible. LO- 30x24x26 mm with follicles up to 15 mm, RO-35x30x30 mm with GF-up to 21 mm Endom 9 mm I phase of cycle	322	126.2	5.63		E2-63.3 RLH-26.89		5.7	86	normal
13 y	6.25 + 5+5 (11.77 mg/m2)	50 + 50	120/63	–	TH V PIV A(+) Regular menses	RO- cyst 32x28x25 mm LO—cyst 47x30x35 mm									
13 y 2 m	6.25 + 5+5 (11.77 mg/m2)	50 + 50	112/74	–	TH V PIV A(+) Regular menses	LO- 31x22.1mm with follicles up to 19.3 mm, RO-37x22.4 mm with GF-up to 18.2 mm Endom 4.5 mm Duphaston 2 x tbl since 16 day of cycle for 10 days every month									
13 y 6 m							1177.4	121.0					5.99		K – 5.19 Cl – 99.5

ACTH, Adrenocorticotropic Hormone; Cort., Cortisol; 17OHP, 17 Hydroxyprogesterone; TST, Testosterone; A, Androstendione; E2, Estradiol; FSH, Follicle Stimulating Hormone; LH, Luteinizing Hormone; DHEA S, Dehydroepiandrosterone Sulfate; PRA, Plasma Renin Activity; Aldos., Aldosterone; Na, Sodium; K, Potassium; Cl, Chloride; mIU, Milli international Units; mcg, Micrograms; mmol, Millimoles; l, Liter; m, Months; y, Years; GF, Graafian follicles; Th, gr.thelarche, ang.breast, P, gr.pubarche, ang.pubic hair, A, gr.axillarche, ang.axillary hair; the mark> means the laboratory did not perform further dilution of the sample, and therefore, we do not have the exact value beyond this upper limit.

It includes the daily hydrocortisone (HC) dosing regimen—given both as the absolute daily dose (in mg) and as a calculated dose normalized to body surface area (mg/m²)—along with fludrocortisone (FC) supplementation (in mcg). Additionally, the table reports blood pressure (BP, in mmHg), bone age assessments according to the Greulich-Pyle (GP) method, pubertal staging, and ultrasound (US) findings of the adrenal glands and ovaries.

### 2.2 Biochemical and hormonal parameters

Biochemical and hormonal parameters were routinely analyzed in the Department of Biochemistry of the University Children Hospital in Krakow.

### 2.3 24-hour analysis of steroid profile in urine

24-hour analysis of steroid profile in urine was performed in the Department of Clinical Biochemistry of The Children’s Memorial Health Institute in Warsaw (**Table 5**). The analysis was performed using a Hewlett-Packard HP 6890 Series GC System gas chromatograph equipped with a Hewlett-Packard 5973 Mass Selective Detector and a 12-meter HP Ultra 1 fused silica capillary column (Hewlett-Packard). Peak identification was based on comparison of the retention times of the observed peaks with those of steroid standards. Quantitative calculations were performed by comparing the peak areas of the detected steroid standards with the area of the internal standard peak (stigmasterol). Following GC/MS analysis of the sample, a chromatogram is obtained, which graphically represents the detector signal intensity as a function of retention time. The resulting mass spectrum is characteristic of a specific chemical compound. The mass spectrometer is synchronized with computer-based data processing software, enabling comparison of the acquired mass spectrum with a reference library of known mass spectral patterns corresponding to compounds with established chemical structures. Using this GC-MS technique—designed for the separation and identification of mixture components—a steroid profile analysis was conducted, encompassing 38 steroid metabolites (**Table 5**). In cases where precise separation of compound mixtures proves challenging, the method of selected ion monitoring (SIM) is employed. The SIM technique offers substantially greater sensitivity and selectivity compared to full-scan acquisition, which detects all ions resulting from the fragmentation of a given chemical compound.

**Table 5:** Urine steroid profile signatures for Cases 1-3.

Steroid profile	Value [ug/24h]	Norm	Steroid profile	Value [ug/24h]	Norm	Steroid profile	Value [ug/24h]	Norm
Case 1	F 14 days old		Case 2	M 8 days old		Case 3	F 4 months old	
AN	25.5	(1-10)	AN	24.2	(1-10)	AN	1735.5	(<20)
ET	1.6	(1-5)	ET	1	(1-5)	ET	123.3	(<20)
11-OAN/ET	7.5	(5-20)	11-OAN/ET	5	(5-20)	11-OAN/ET	24.1	(5-20)
11-OHAN	15.1	(5-20)	11-OHAN	31	(2-20)	11-OHAN	826.3	(2-20)
11-OHET			11-OHET	1	(<20)		0	(<20)
ET/AN	0.1		ET/AN	0		ET/AN		
DHA	14.7	(1-10)	DHA	17.8	(1-10)	DHA	1252.9	(<20)
5-AND	15.2	(1-10)	5-AND	20.6	(1-10)	5-AND	136.4	(<20)
16a-OHDHA	4752.3	(250-1250)	16a-OHDHA	1280.5	(135-500)	16a-OHDHA	10405.1	(250-1250)
An-3-ol	193.2	(40-600)	An-3-ol	201.4	(40-600)	An-3-ol	4182.5	(<20)
5-PT	75.6	(2-20)	5-PT	17.4	(2-20)	5-PT	8173.3	(2-20)
16-OHPN	2114.8	(195-1295)	16-OHPN	2043.1	(110-495)	16-OHPN	6882	(110-495)
17-OHPN(5beta)	92.9	(5-20)	17-OHPN(5beta)	7.7	(4-19)	17-OHPN(5beta)	890	(5-20)
17-OHPN(5alfa)	1.5	(<20)	17-OHPN(5alfa)	3.1	(<20)	17-OHPN(5alfa)	603.1	(<20)
PT	109.1	(5-20)	PT	5.8	(5-21)	PT	378.1	(5-20)
PTN	8.3	(0-5)	PTN	5	(0-5)	PTN	108.1	(0-5)
PD	21.6	(2-20)	PD	6.2	(2-20)	PD	92.8	(2-20)
E1			E1	0		E1	0	
E2			E2	0		E2	0	
E3			E3	0		E3	0	
THS	9.5	(1-3)	THS	5.8	(1-3)	THS	45.7	(1-3)
THDOC	0	(2-38)	THDOC	0	(2-38)	THDOC	0	(2-38)
THA	29.5	(5-30)	THA	19.3	(5-30)	THA	0	(5-30)
allo-THA	15.1	(15-90)	allo-THA	10.1	(15-90)	allo-THA	0	(15-90)
THB	0	(4-55)	THB	0	(4-55)	THB	0	(4-55)
allo-THB	0	(50-130)	allo-THB	0	(50-130)	allo-THB	0	(50-130)
THAldo	28.3	(4-12)	THAldo	2.2	(4-12)	THAldo	0	(4-12)
THE	11.5	(5-50)	THE	61.5	(38-408)	THE	92.5	(38-408)
THF			THF	5.1	(100-500)	THF	47.2	(100-500)
allo-THF			allo-THF	5.3	(115-680)	allo-THF	296.1	(115-680)
THF/allo-THF			THF/allo-THF	1		THF/allo-THF	0.16	
THF+allo-THF/THE			THF+allo-THF/THE	0.2	(0.7-1.3)	THF+allo-THF/THE	3.71	(0,7-1,3)
a-CTN	20.9	(20-100)	a-CTN	24.5	(20-100)	a-CTN	51	(20-100)
b-CTN	49.3	(20-100)	b-CTN	9.1	(20-100)	b-CTN	49.5	(20-100)
b-CT	15.3	(5-20)	b-CT	21.4	(5-20)	b-CT	206.4	(5-20)
a-CT	19	(5-20)	a-CT	89.6	(5-20)	a-CT	461.7	(5-20)
E	14.6	(5-20)	E	45.4	(5-20)	E	42.3	(5-20)
F	7.8	(3-20)	F	9.6	(3-20)	F	40	(5-20)
F/E	0.5	(0.34-0.74)	F/E	0.2	(0.34-0.74)	0.5	0.94	(0,34-0,74)
6b-OHF	0		6b-OHF	0		6b-OHF	0	
20a-DHF	0		20a-DHF	0		20a-DHF	26	

AN, Androsterone; ET, Etiocholanolone; 11-OHAN, 11-OH- Androsterone; 11-OHET, 11-OH- Etiocholanolone; DHA, Dehydroepiandrosterone; 5AND, 5-Androstenediol; 16a-OHDHA, 16alpha-OH-Dehydroepiandrosterone; An-3-ol – 5, Androstenetriol; 5-PT, 5-Pregnenetriol; 16-OHPN, 16-alpha-OH-pregnenolone; 17-OHPN(5beta), 5-beta 17-OH-Pregnanolone; 17-OHPN(5alfa), 5-alpha 17-OH-Pregnanolone; PT, Pregnanetriol; PTN, Pregnanetriolon; PD, Pregnanediol; E1-, E2-, E3-, THS, Tetrahydro-11-deoxycortisol; THDOC, Tetrahydro-11-deoxycorticosterone; THA, Tetrahydro-11-dehydrocorticosterone; AlloTHA, Allo-tetrahydro-11-dehydrocorticosterone; THB, Tetrahydro-corticosterone; Allo-THB, Allo-tetrahydro-corticosterone; THAldo, Tetrahydro-aldosterone; THE, Tetrahydro-cortisone; THF, Tetrahydro-cortisol; Allo-THF, Allo-tetrahydro-cortisol; a-CTN, Alpha-cortolone; b-CTN, Beta-cortolone; a-CT, Alpha-cortolone; b-CT, Beta-cortolone; E, Cortisone; F, Free cortisol.

### 2.4 The molecular genetic analyses

The molecular genetic analyses for the three cases were performed at different time points in external diagnostic centers, and the change in Illumina sequencer platform reflects internal updates implemented by these centers over time. Unfortunately, this aspect was beyond our control. Nevertheless, in all three cases, next-generation sequencing (NGS) was performed using targeted panels with custom-designed gene-specific primers to minimize off-target amplification and ensure high specificity for the HSD3B2 gene, despite its high sequence homology with HSD3B1 (**Figures 2**-**4**, **Table 6**).

**Table 6:** *HSD3B2* genetic results in described cases.

Cases	Nucleotide position	Protein change	Variant type	Exon	Father	Mother
Case 1	c.760T>G hom	p.Tyr254Asp	missense variant	4	heterozygous	heterozygous
Case 2	c.760T>G	p.Tyr254Asp	missense variant	4	heterozygous	
	c.308-6G>A	N/A	splicing variant	4		heterozygous
Case 3	c.849del hom	N/A	frameshift variant	4	heterozygous	heterozygous

The variants identified in Cases 1 and 2 were confirmed by bidirectional Sanger sequencing using primers specific to HSD3B2, which are designed to avoid amplification of the HSD3B1 paralog (**Figures 2**, **3**). We acknowledge that the quality of the Sanger sequencing chromatograms presented for the parents in **Figure 2** is suboptimal. Unfortunately, we do not have access to alternative or higher-quality chromatograms, as the parental DNA samples were collected in early 2020 and 2024, respectively, and are no longer available for repeat analysis. In Case 3, the family did not attend follow-up appointments necessary to obtain a separate sample for confirmatory testing. Therefore, Sanger sequencing could not be completed in that case (**Figure 4**).

In case 1 and 3 the study of the *HSD3B2* gene involved the analysis of coding exon sequences (including 10–20 nucleotide intronic flanking regions). The enriched DNA regions were sequenced using in case 1 the NovaSeq4000 sequencer (Illumina) with a read length of 2x151 nucleotides. Genetic variants were identified using the Burrows-Wheeler Aligner. The average sequencing depth was 157.0 with a quality threshold of 98.7%. The NM_000198.4(*HSD3B2*): c.760T>G (p.Tyr254Asp) was revealed. In case 3 the enriched DNA regions were sequenced using the NovaSeq500 sequencer (Illumina) with a read length of 2x150 nucleotides. Genetic variants were identified using the Burrows-Wheeler Aligner. The average sequencing depth was 94.1 with a quality threshold of 100% sequencing. The NM_000198.4(*HSD3B2*): c.849delG (p.Trp283fs) was revealed. In case 2 sample panel NGS covering genes connected with abnormal genital development was analyzed using the oligonucleotide-selective sequencing (OS-Seq™) (PMID: 22020387) NGS method on the NextSeq sequencing system (Illumina). *HSD3B2* c.308-6G>A and c.760T>G, p.(Tyr254Asp) were confirmed using bidirectional Sanger. The classification of variants was based on the guidelines developed by the American College of Medical Genetics and Genomics and the American Association for Molecular Pathology (17). Exclusively pathogenic and potentially pathogenic variants were reported based on the outlined criteria.

### 2.5 A literature overview

A literature overview has been conducted using the PubMed and Embase databases in order to gather previously described 3β-HSD2D cases and identify variants in the *HSD3B2* gene.

### 2.6 Ethics

This study was approved by the relevant institutional review board (The Ethics Committee of the Jagiellonian University opinion number:1072.6120.120.2022 issued on 14th December 2022). Written informed consent was obtained from all participants and/or their parents. Written informed consent was obtained from the individual(s) and minor(s) legal guardian/next of kin for the publication of any potentially identifiable images or data included in this article.

## 3 Case presentation

### 3.1 Case 1

A female neonate was delivered at 36 weeks of gestation via elective cesarean section due to a history of three previous cesarean sections. Prenatal care included corticosteroid administration at 34 weeks’ gestation for fetal lung maturity. The infant’s birth weight was 2,880 grams and Apgar scores were of 5, 6, 8, and 8 at 1, 3, 5, and 10 minutes, respectively. The family history was significant for a sibling who died in the neonatal period due to DiGeorge syndrome associated with an interrupted aortic arch. At birth, the neonate required resuscitation with positive pressure ventilation due to respiratory distress. She initially received inflations with a resuscitation bag, followed by non-invasive respiratory support with nasal continuous positive airway pressure (nCPAP). Physical examination revealed facial dysmorphic features, and a cardiac murmur graded 2/6 on the Levine scale. Initial laboratory evaluations showed no significant abnormalities, and infection markers were negative. An echocardiogram revealed a right-sided aortic arch and a large perimembranous ventricular septal defect (VSD) measuring 4–6 mm with left-to-right shunting and a gradient of approximately 60 mmHg. The VSD was partially restricted by tricuspid valve tissue. There was also evidence of a vascular ring due to an aberrant left subclavian artery with a retroesophageal course. The left subclavian artery had proximal stenosis. On the 5^th^ day of life due to the progressing skin hyperpigmentation and family history, endocrine evaluation was initiated. Physical examination noted a mild clitoromegaly and hyperpigmentation of the labia majora (Prader II). Laboratory tests revealed significant electrolyte imbalances including decreased sodium levels from 135 mmol/L to as low as 127 mmol/L (N: 136–146 mmol/L), increased potassium concentration up to 6.1 mmol/L (N: 3.5-5.1 mmol/L) and decreased chloride - 93 mmol/L (N: 101–109 mmol/L).

Hormonal results indicated adrenal insufficiency, with an initial ACTH level of 1,394 pg/mL (N: 7.2-63.3 pg/mL), low cortisol level of 5.0 μg/dL (N: 3.7-19.4 μg/dL) and elevated 17-OHP (94.75 ng/mL; N: 2.4-16.8 ng/mL), as well as androstenedione (>10 ng/mL; N: 0.30-3.32 ng/mL).

Based on the clinical presentation and elevated 17OHP concentrations, the initial suspected diagnosis was congenital adrenal hyperplasia (CAH) due to 21-hydroxylase deficiency. In particular, the presence of electrolyte disturbances (hyponatremia and hyperkalemia), together with markedly elevated 17OHP levels, was consistent with classic salt-wasting 21-hydroxylase deficiency and led to this preliminary working diagnosis. At 11 days of age, due to persistent vomiting, diarrhea, and worsening electrolyte imbalances, hydrocortisone therapy was initiated intravenously at the dose of 39.5 mg/m2, later switched to oral administration. Fludrocortisone was added on day 16 to address mineralocorticoid deficiency. Sodium supplementation with 10% NaCl solution was administered orally to correct hyponatremia. The treatment led to the stabilization of electrolyte levels and an improvement in skin pigmentation. The neonate exhibited an ineffective sucking reflex and inadequate weight gain. Enteral nutrition was provided via a nasogastric tube while conducting oral stimulation therapy. Gradually, she transitioned to feeding with a bottle, consuming fortified breast milk with human milk fortifier, and demonstrated steady weight gain.

The urinary steroid profile collected at the age of 14 days was consistent with the biochemical signature of HSD3B2 deficiency (**Table 5** **).** The 24-hour urine analysis revealed elevated levels of 5-pregnenetriol (5PT), the primary urinary metabolite of 17α-hydroxypregnenolone, along with increased excretion of DHEA, pregnanetriol (PT), 17-hydroxyprogesterone (17OHP), and pregnanediol (PD). These findings reflect accumulation of Δ5 steroid precursors due to impaired Δ5–Δ4 conversion. The elevations in PT, PD, and 17OHP are explained by the action of peripheral HSD3B1, which is expressed in the placenta and peripheral tissues such as the liver, and can convert accumulating Δ5 steroids (e.g., 17OHPreg and DHEA) into Δ4 derivatives, including 17OHP and downstream metabolites. Importantly, the 5PT/pregnanetriolone (PTONE) ratio was 9.0, which is markedly elevated compared to values typically observed in CYP21A2 deficiency and aligns with the diagnostic pattern expected in HSD3B2 deficiency. Taken together, these findings supported the diagnosis of HSD3B2 deficiency in this case.

Due to signs of congestive heart failure, including tachypnea and hepatomegaly, the patient was started on furosemide, digoxin, and later hydrochlorothiazide. At 8 weeks of age, she was transferred to a tertiary cardiac center for surgical intervention. On two months of age, she underwent surgical closure of the VSD with a Dacron patch, tricuspid valve repair, and release of the vascular ring formed by the aberrant left subclavian artery. Intraoperative management included stress-dose hydrocortisone (50–100 mg/m² intravenously) to address adrenal insufficiency during the surgery. The postoperative period was complicated by anemia, requiring a transfusion of 50 mL packed red blood cells. Regular monitoring showed gradual improvement in cardiac function. Echocardiography post-surgery revealed residual VSDs measuring approximately 1–2 mm with minimal left-to-right shunting and good ventricular function (ejection fraction of 71%).

The patient was referred for genetic analysis, where a targeted next-generation sequencing panel for CAH was performed (**Figure 2** **).** Described previously in literature, a homozygous missense pathogenic variant c.760T>G (p.Tyr254Asp) in exon 4 of the *HSD3B2* gene was identified. Sanger sequencing confirmed the homozygous pathogenic variant in the patient and heterozygosity in both parents, indicating autosomal recessive inheritance (**Figure 2** **).**

Under hydrocortisone and fludrocortisone therapy, the patient’s electrolyte balance stabilized; however, due to overtreatment in the first months of life, growth velocity decreased and weight gain accelerated (**Table 2**, **Figure 6**). Regular endocrinological and cardiological follow-ups were initiated. At her most recent evaluation, the patient remains clinically stable on adjusted doses of hydrocortisone and fludrocortisone (**Table 2**). Despite improved hormonal control and dose reduction over time, she continues to present with short stature (height below the 3rd percentile) and an increased weight-for-height ratio, findings consistent with the early period of glucocorticoid overexposure, as illustrated in **Figure 6**.

The girl shows normal psychomotor development, however continues to experience occasional constipation and abdominal bloating, managed with dietary modifications and laxatives. She has been referred to gastroenterology and nephrology for further evaluation to identify potential causes for her growth delay, with celiac disease already excluded.

### 3.2 Case 2

The male patient was born to healthy, non-consanguineous parents following a pregnancy complicated by gestational diabetes mellitus, which was managed with insulin therapy. He was the third pregnancy but the first live birth, delivered at 39 weeks of gestation via spontaneous vaginal delivery. At birth, he had a weight of 3,750 g, a length of 56 cm, and Apgar scores of 9 at both 1 and 5 minutes.

At delivery, the neonate exhibited atypical genitalia, including a penis measuring 2.7 cm in length with proximal perineal hypospadias, characterized by a urethral meatus located within the urogenital sinus. Additional findings included a hyperpigmented, bifid scrotum with palpable gonads bilaterally within the scrotal sacs. His skin displayed jaundiced discoloration with a bronze hue, most pronounced over the lower abdomen and urogenital region. No facial dysmorphisms or other congenital anomalies were observed.

At the age of 5 days, he was referred to the tertiary DSD unit. Electrolyte measurements revealed a sodium level of 140 mmol/L, a slightly elevated potassium level of 6.06 mmol/L, and normal chloride and calcium levels. His blood pressure was normal. Ultrasound examinations revealed enlarged and convoluted adrenal glands (right adrenal up to 40 mm, left adrenal up to 34 mm). Both testes and epididymides were visualized, with communicating hydroceles in the inguinal canals, and uterus was absent. Laboratory tests were performed and confirmed a karyotype of 46, XY, indicating male genetic sex.

Hormonal evaluation revealed markedly elevated ACTH (547.4 pg/mL) and DHEA-S (>1,500 μg/dL), while cortisol levels were within the upper normal range. However, adrenal reserve was not assessed by a Synacthen test, and hydrocortisone therapy was not initiated at that time. Testosterone levels were appropriate for age. In this 46,XY patient, the measured estradiol concentration was 274.1 pg/mL -an unusually high value for a neonate with a male karyotype. However, we interpret this finding in the context of fetal adrenal physiology. Specifically, this patient demonstrated significantly elevated levels of 16α-hydroxy-DHEA (16α-OH-DHEA), a steroid produced predominantly by the fetal adrenal zone. The fetal zone is highly active during late gestation and is a major source of DHEA and its hydroxylated derivatives. These Δ5 precursors can be converted in peripheral tissues-particularly in the placenta and fetal liver- into estrogens, including estradiol, via aromatization. Thus, we attribute the elevated estradiol to increased substrate availability from the persistent fetal adrenal zone, rather than to gonadal or pathological estrogen production.

The urinary steroid profile assessed at the age of 8 days excluded deficiencies of 21-hydroxylase, 17α-hydroxylase, and 5α-reductase, but increased excretion of metabolites from the fetal zone of the adrenal cortex was noted (**Table 5** **).** This profile was not typical for HSD3B2 deficiency. The 24-hour urine analysis showed normal levels of 5PT, DHEA, PT, 17OHP, and PD, indicating no significant accumulation of Δ5 precursors or their metabolites. Furthermore, the 5PT/PTONE ratio was 3.5, a value that does not meet the threshold typically seen in HSD3B2 deficiency and does not clearly distinguish this case from other forms of congenital adrenal hyperplasia. As a result, the urinary steroid pattern was considered inconclusive, and the patient was referred for molecular genetic testing to clarify the underlying etiology.

At the age of 6 months CAH due to 3β-HSD2D was confirmed (**Figure 3**). Genetic analysis, using next-generation sequencing on a panel of 39 genes associated with disorders of sex development, identified two variants in the *HSD3B2* gene: a novel splice site variant c.308-6G>A, predicted to affect mRNA splicing by *in vitro* analyses and a missense pathogenic variant c.760T>G (p.Tyr254Asp). Both were confirmed with Sanger sequencing and were detected in patient’s parents in heterozygosity (**Figure 3**). The clinical, hormonal, and genetic findings, lead to the final diagnosis of 3β-HSD2D. Despite the enzymatic deficiency, the patient did not exhibit signs of clinical adrenal insufficiency, and glucocorticoid therapy was not introduced. The family was educated on recognizing signs of adrenal crisis and the importance of stress dosing with hydrocortisone during periods of illness or surgery.

The urologist confirmed the diagnosis of perineal hypospadias with bifid scrotum and micropenis. As shown in **Table 3**, between weeks 35 and 37 of life, the patient received three intramuscular doses of 20 mg testosterone enanthate to enhance the surgical field for the urological procedure. Management included staged surgical interventions for the urogenital anomalies. At six months of age, the patient underwent the first stage of surgical correction for perineal hypospadias using the onlay island flap technique and correction of penile curvature. A second-stage surgery was performed at one year of age to repair a urethral diverticulum and complete the urethroplasty. At seven years, orchiopexy was performed for left-sided canalicular cryptorchidism. During all surgical procedures, perioperatively, the patient received hydrocortisone intravenously.

At the age of 1 year and 10 months, the hydrocortisone regimen was introduced due to a rapid growth velocity of 15.9 cm/year and acceleration of bone age as presented in **Table 3**, **Figure 6**. Since the age of 3.5 years the patient has been referred to a speech therapist, due to the delayed speech development. **Table 3** presents results of longitudinal hormonal assessments of the patient.

### 3.3 Case 3

A female infant was born at 39 weeks of gestation via vaginal delivery with a birth weight of 2600 g, length 52 cm, head circumference 34 cm and an Apgar score of 10 at both one and five minutes. At four months of age, the infant presented with increased perspiration, poor feeding, and failure to thrive over the preceding months. Upon admission, her weight was 4,710 grams and physical examination revealed pale, mottled skin and slightly decreased muscle tone. Notably, there were signs of virilization of the external genitalia, including an enlarged clitoris (Prader II). Laboratory tests showed significant electrolyte imbalances: hyponatremia (sodium 120 mmol/L), hyperkalemia (potassium 7.70 mmol/L), and hypochloremia (chloride 90 mmol/L). Renal function parameters showed elevated urea (9.6 mmol/L) and creatinine (40.6 μmol/L) concentrations. Hormonal assays were performed revealing elevated ACTH (1,282 pg/mL), 17-hydroxyprogesterone (>9.50 ng/mL), DHEA-S (>822 μg/dL, unfortunately, the laboratory did not perform further dilution of the sample, and therefore, we do not have the exact value beyond this upper limit), and normal cortisol (162.2 ng/mL). An abdominal ultrasound demonstrated significantly enlarged adrenal glands and kidneys with numerous small cysts located in the pyramids, suggestive of polycystic kidney disease (**Table 4**).

The urinary steroid profile assessed at the age of 4 months was highly characteristic of HSD3B2 deficiency (**Table 5**, **Figure 7**). The 24-hour urine collection revealed marked elevation of 5PT, the principal metabolite of 17α-hydroxypregnenolone, along with significantly increased excretion of DHEA, PT, 17OHP, and PD. These elevations reflect the accumulation of Δ5 steroid precursors due to impaired Δ5–Δ4 isomerization. As in Case 1, the increased urinary excretion of PT, PD, and 17OHP likely results from peripheral conversion of Δ5 steroids by HSD3B1, which is active in the placenta and peripheral tissues and contributes to the formation of Δ4 steroids despite the enzymatic block in the adrenal glands. Notably, the 5PT/PTONE ratio was 75.6- substantially above values observed in CYP21A2 deficiency and strongly indicative of HSD3B2 deficiency. This combination of findings provided compelling biochemical evidence in support of the diagnosis.

CAH due to 3β-HSD2 deficiency was confirmed by genetic testing which revealed null pathogenic variant c.849del (p.Trp283*) in homozygosity in the 4th exon of the *HSD3B2* gene (**Figure 4**). The patient was put on a course of hydrocortisone and fludrocortisone therapy leading to clinical improvement and normalization of electrolyte levels (**Table 4**).

Following the glucocorticoid therapy the patients showed a tendency to increased calcium levels and has been referred to a nephrologist. This might have manifested as a complication following high doses of HC, which were required due to frequent upper respiratory tract infections.

During a control visit at the age of 7 months the disappearance of renal cysts was noted, and the structure of kidneys appeared normal. At the age of seven years and two months, the episodes of abdominal pain occurring mainly in the evenings, resolving spontaneously or after bowel movements have been reported. Physical examination was unremarkable, and lab tests were within normal limits. An abdominal USG revealed a gallbladder containing a 3.5 mm echogenic structure with a weak acoustic shadow, consistent with a gallstone, leading to a diagnosis of cholelithiasis without signs of inflammation. Treatment with ursodeoxycholic acid was initiated, with recommendary dietary modifications. Over the following year, she continued to experience intermittent abdominal pain. At eight years old, she performed a hydrogen breath test with lactulose that indicated small intestinal bacterial overgrowth (SIBO) treated with metronidazole for ten days, followed by a probiotic regimen. Despite initial improvement, abdominal pain recurred even though subsequent hydrogen breath test with lactose was negative, ruling out lactose intolerance. Further gastrointestinal evaluation did not reveal additional pathology. The recurrent abdominal pain and diagnosis of SIBO suggested that gastrointestinal dysmotility or altered gut flora might be contributing factors. Pubertal development commenced at 7 years and 3 months of age, with thelarche at Tanner stage II observed. Consequently, the hydrocortisone dose was increased to slow the progression of puberty. Suppression with a GnRH analogue was not initiated, as the bone age remained within normal limits (advanced by no more than one year relative to chronological age). Menarche occurred at 10 years and 7 months. Initially, menstrual cycles were irregular and heavy, necessitating gynecological follow-up. Conservative management was implemented with tranexamic acid (Exacyl) and etamsylate (Cyclonamine). A follow-up pelvic ultrasound revealed a 4 cm ovarian cyst, prompting the initiation of dydrogesterone therapy from day 16^th^ of the menstrual cycle for 10 days each month. As presented in the **Table 4**, dydrogesterone has been effective in controlling ovarian cysts. However, in our patient, ovarian cysts have recurred alternately in both ovaries, reaching sizes of up to 5 cm (**Figure 5**). If this issue persists, we plan to initiate treatment with an oral contraceptive pill.

At the most recent follow-up, at the age of 13 years and 8 months, the patient continues to receive hydrocortisone (6.25 mg in the morning, 5 mg at midday, and 5 mg in the evening) and fludrocortisone therapy (0.05 mg twice daily). The observed growth rate of 2.1 cm/year is consistent with a post-menarcheal adolescent approaching final height and reflects the natural deceleration in growth following the pubertal growth spurt (**Figure 6**). Her physical examination revealed a normosthenic build with proportional growth, embracing the height of 151 cm. She was in thelarche stage V and pubarche stage V according to Tanner staging, with axillary hair present. Menses are regular. Acne lesions were noted on her face and chest. Hormonal assessments showed elevated 17-hydroxyprogesterone levels, with adrenocorticotropic hormone levels within the target range under her current therapy, cortisol levels appropriate for her hydrocortisone dosing schedule, and estradiol levels consistent with her pubertal status. She continues to receive gynecological care, including ongoing dydrogesterone therapy to compensate for progesterone deficiency. Gastroenterological care involves monitoring her gallstones with regular follow-up, however surgical intervention was deferred due to the absence of symptoms. Currently she remains on ursodeoxycholic acid for gallstone management. Additionally, dermatological care was initiated for acne vulgaris.

## 4 Discussion

### 4.1 Overview

This report presents a long follow-up of three cases of 3β-HSD2 deficiency, involving two female infants with classic salt wasting forms and one male infant with classic simple virilizing form of this type of congenital adrenal hyperplasia, all of whom exhibited symptoms during early infancy. Two affected female patients presented with adrenal insufficiency, resulting in clinical manifestations such as poor feeding, vomiting, diarrhoea, failure to thrive, hyperkalemia, and hyponatremia and mild virilization of external genitalia. They also demonstrated high ACTH levels, which caused increased melanocyte-stimulating hormone activity, leading to hyperpigmentation of the skin, areola and external genitalia. The male patient presented signs of undervirilisation with progressive GnRH independent precocious puberty starting after the first year of age.

Deficiency of 3β-hydroxysteroid dehydrogenase type 2 (3β-HSD2) leads to disruptions in both adrenal and gonadal steroidogenesis (**Figure 1**). This should theoretically result in elevated levels of precursor Δ5 steroids - including pregnenolone, 17-hydroxypregnenolone, dehydroepiandrosterone (DHEA), and androstenediol, while concentrations of downstream metabolites such as progesterone, 17-hydroxyprogesterone (17-OHP), androstenedione, and testosterone should be diminished (**Figure 7A**) (18). However, in our patients and in broader clinical practice, urinary metabolites of 17-OHP and testosterone are found to be elevated. In case 1, the neonatal 17OHP concentrations of 187 and 330 nmol/L were measured on the second and third day of life in a newborn delivered at 36 weeks of gestation. These values were obtained using a fluoroimmunoassay (FIA), which is the standard method employed in the Polish national newborn screening program for congenital adrenal hyperplasia (CAH). According to method-specific and gestational age-adjusted reference ranges for FIA, the threshold 17OHP value for infants born at 36 weeks is <85.5 nmol/L on day 2 of life and <75 nmol/L on day 3. The reported values are therefore clearly elevated relative to these cutoffs.

The presence of elevated serum 17OHP in the context of presumed HSD3B2 deficiency may initially seem paradoxical. However, this finding can be explained by peripheral conversion of Δ5 steroid precursors to their Δ4 counterparts. Tissues such as the placenta and liver express the HSD3B1 isoform, which is capable of converting accumulated Δ5 steroids-such as 17OH-pregnenolone and DHEA-into Δ4 steroids, including 17OHP. This extra-adrenal enzymatic activity may therefore account for elevated circulating 17OHP levels despite impaired adrenal 3β-HSD2 function.

The persistence of the fetal adrenal zone may further contribute to increased steroid precursor production in the neonatal period, particularly in a preterm infant born at 36 weeks’ gestation. This interpretation is supported by the steroid profile shown in **Table 5**, which includes elevated levels of 16α-hydroxy-DHEA (16α-OH-DHEA), a steroid derived predominantly from the fetal zone of the adrenal cortex. The fetal adrenal zone is characterized by robust expression of CYP17A1, which drives DHEA synthesis. In addition, the enzyme CYP3A7, highly expressed in the fetal adrenal and placenta, catalyzes the 16α-hydroxylation of DHEA to 16α-OH-DHEA. Fetal zone produces large amounts of Δ5 steroids, not Δ4 steroids. However, these Δ5 steroids (like DHEA and 17OH-pregnenolone) can be converted in peripheral tissues via HSD3B1, contributing indirectly to serum 17OHP. So while the fetal zone itself does not directly produce 17OHP, it contributes a high load of precursors that may be peripherally converted to 17OHP.

It is indeed biochemically and physiologically plausible that peripheral HSD3B1 activity and increased Δ5 steroid production from the fetal adrenal zone together explain elevated serum 17OHP in a neonate with 3β-HSD2 deficiency—even under hydrocortisone treatment. However, it’s important to acknowledge that this is indirect 17OHP production, not adrenal in origin. Hydrocortisone might suppress adrenal output but not peripheral conversion. RIA cross-reactivity may slightly exaggerate true 17OHP levels.

The rise in testosterone levels may also result from peripheral conversion of excess dehydroepiandrosterone (DHEA) into testosterone by 3β-HSD1 or from the subsequent transformation of DHEA into testosterone via elevated 17-OHP levels, facilitated by enzymes such as 17,20-lyase (19). Additionally, in the urinary steroid profile of the patient from Case 3, we also found some increased glucocorticoid metabolites (THS, a-CT, b-CT, F, E), while others were decreased (THF, allo-THF, THE, a-CTN, b-CTN) (**Figure 7B**). This could have been the result of severe accumulation of 17-OHP, converted from 17-hydroxypregnenolone by 3β-HSD1, stimulated by markedly elevated ACTH levels (1,394 pg/mL). Nonetheless, the clinical presentation was consistent with classical salt-wasting syndrome, supported by markedly low urinary levels of mineralocorticoid metabolites. Consequently, hydrocortisone (HC) and fludrocortisone (FC) supplementation had to be initiated. Interestingly, in Case 2, despite predominantly decreased mineralocorticoid metabolites (**Figure 7C**), fludrocortisone (FC) supplementation was not required, as plasma renin activity remained within the normal range, along with normal electrolyte levels and blood pressure.

The clinical presentation of the disease also depends on the residual activity of 3β-HSD2. When enzyme activity is below 1–2%, the classic form manifests with adrenal insufficiency, including both aldosterone and glucocorticoid deficiencies, along with androgen excess in females and androgen deficiency in males (**Figure 7D**). However, research suggests that if enzyme activity is equal to or exceeds 2%, the condition presents as the classic virilizing form, characterized primarily by virilization, with little to no glucocorticoid or mineralocorticoid deficiency (20).

The clinical and biochemical variability between our patients demonstrates first-hand the intricate and unexpected manifestations of 3β-HSD2D, which pose significant diagnostic difficulty and underline the importance of genetic testing.

### 4.2 Physiology

From the perspective of a pediatrician and a pediatric urologist managing patients with 3β-HSD2 deficiency, it is essential to understand the underlying mechanisms leading to incomplete masculinization in male neonates and virilization in female neonates with this condition.

Recent studies have revealed clinically significant changes in the concentrations of enzymes regulating steroidogenesis during fetal development. In the fetal zone (FZ) of the adrenal cortex, androgen biosynthesis pathways predominate, whereas the capacity for cortisol and aldosterone synthesis in the definitive zone (DZ) develops progressively. Adrenal steroidogenesis begins around the 7th gestational week (GW). Between the 8th and 9th GW, the presence of 3β-hydroxysteroid dehydrogenase type 2 (3β-HSD2), has been detected in the DZ of both sexes. By the 8th GW, cortisol can be identified in the adrenal glands, and the cortisol–ACTH feedback loop begins to establish. After the 9th GW, cortisol synthesis declines, and 3β-HSD2 becomes undetectable after the 14th GW. Its activity gradually increases again from the 19th GW onward. A transient rise in cortisol production between the 8th and 13th GW—corresponding to the *masculinization programming window* (MPW)—is critical for female fetuses, as cortisol suppresses ACTH, thereby inhibiting adrenal androstenedione production (which would otherwise be converted to testosterone). This suppression prevents virilization of the external genitalia in healthy female fetuses. Simultaneously, testosterone is produced in the fetal testes, which is essential for normal male genital development.

Female fetuses with impaired adrenal steroidogenesis—such as those with congenital adrenal hyperplasia (CAH) due to 21-hydroxylase deficiency—may produce excessive adrenal androgens due to a lack of ACTH suppression. This includes potent androgens such as 11-ketotestosterone, which can result in complete virilization of the external genitalia by the 12th–13th GW. In 3β-HSD2 deficiency, genital anomalies in female fetuses are similarly explained by the low activity of 3β-HSD2 during the critical window of external genital development (21). As presented by Gotto et al. in humans, early cortisol biosynthesis provides a mechanism to safeguard female sexual development (22). In female fetuses, the absence of adequate testosterone levels in early gestation leads to Wolffian duct regression, while the Müllerian duct differentiates into the fallopian tubes and uterus (23). During the third trimester, 3β-HSD1 contributes to the conversion of DHEA into testosterone. Elevated androgen levels at this stage can lead to varying degrees of virilization in affected females, manifesting as clitoromegaly and, in some cases, partial labial fusion (24).

The deficiency of 3β-HSD2 in the testes disrupts androgen biosynthesis, resulting in genital abnormalities such as micropenis, hypospadias, and severe underdevelopment of the external genitalia (25–38). Male infants with this condition may exhibit impaired testosterone synthesis during early fetal development, as androgen production in the fetal testes appears to be significantly higher than that of the adrenal glands. The additional contribution of adrenal-derived dehydroepiandrosterone sulfate (DHEA-S) seems insufficient to compensate for the overall deficit in testosterone. Additionally, the activity of 3β-HSD1 surpasses that of 3β-HSD2, with the latter being most active during the third trimester of pregnancy, after the completion of genital development (13). Although 3β-HSD1 facilitates the conversion of excess dehydroepiandrosterone (DHEA) into testosterone, individuals with a 46,XY karyotype and severe 3β-HSD2 deficiency do not produce sufficient androgens for normal genital development.

### 4.3 Genetics

In the presented cases, distinct phenotypic variability was observed, which could be in part influenced by the specific pathogenic variants and allele configurations in the *HSD3B2* gene (**Table 6**). Each child carried compound heterozygous or homozygous pathogenic variants, though the specific variants differed. The identified pathogenic variants included: nonsense- frameshift mutation, splice-site, and missense variants. All were located in the 4^th^ exon. Kinetic analyses of mutant HSD3B2 proteins associated with both salt-wasting and non-salt-wasting forms of the disease have demonstrated a 4- to 40-fold-or greater-reduction in catalytic efficiency for the conversion of pregnenolone to progesterone or DHEA to androstenedione, depending on the specific mutation and substrate, as reported by Moisan et al. (36).

The increased instability of mutant proteins in individuals with salt-wasting disease, compared to those with the non-salt-wasting form, partially explains the different clinical phenotypes (36). An attenuated or late-onset form of 3β-HSD2 deficiency, identified through steroid measurements, has also been documented (14, 26, 27, 36, 38). Potential pathogenic variants in the distal promoter, polymorphisms or other epigenetic factors affecting enzyme expression cannot be ruled out (14, 26, 27, 36, 38). The observed reduction in 3β-HSD2 activity might also result from changes in the membrane environment that impact catalytic activity or from posttranslational modifications that reduce enzyme function (14, 26, 27, 36). It can be inferred that the transcriptional regulators driving the increased expression of *HSD3B2* may include *NR5A1, NR4A1 (NURR77)*, and GATA6 (21, 28).

The first patient was diagnosed with a NM_000198.4(*HSD3B2)*: c.760T>G (p.Tyr254Asp) variant, which causes a missense change involving the alteration of a conserved nucleotide. The variant allele was found at a frequency of 0.00000479 in 1,461,816 control chromosomes in the GnomAD database, with no homozygous occurrence. In-silico tool predicts a pathogenic outcome for this variant. No clinical diagnostic laboratories have submitted clinical-significance assessments for this variant to ClinVar. Variant is believed to be likely pathogenic due to (PP3, PM2, PP2, PP5) ACMG criteria. Another nucleotide change resulting in same amino acid change has been previously reported as Likely pathogenic in UniProt (11). Notably the same mutation, albeit in a heterozygotic variant, has been previously described by Sanchez et al. in a female patient of Polish descent diagnosed with 3β-HSD2D, who presented with severe acne, hirsutism and amenorrhea (29, 30). During *in vitro* testing of the c.760T>G mutated 3β-HSD2 enzyme, Sanchez et al. found that it demonstrated no significant enzymatic activity (29). It follows that our patient, as a homozygote, would display a complete lack of 3β-HSD2 activity and suffer from classical SW CAH due to *HSD3B2D*.

Our second patient, with a 46, XY karyotype, was identified with two variants in the *HSD3B2* gene: the previously mentioned missense pathogenic variant c.760T>G (p.Tyr254Asp) and a novel NM_000198.4(*HSD3B2)*: c.308-6G>A variant, which causes a splice region, intron change involving the alteration of a non-conserved nucleotide. For the c.308-6G>A variant, we used Franklin by Genoox, GeneBe (https://genebe.net/), Varsome, and MutationTaster2021 (https://www.genecascade.org/MutationTaster2021/) to assess potential effects on splicing and pathogenicity. The variant allele was found at a frequency of 0.0000093 in 1,613,324 control chromosomes in the GnomAD database, with no homozygous occurrence. In-silico tool predicts a benign outcome for this variant. 3/3 splice prediction tools predict alterations to normal splicing. No clinical diagnostic laboratories have submitted clinical-significance assessments for this variant to ClinVar. Variant is classified as VUS (variant of unknown significance due to ACMG criteria (PM2, PP3). In this case the major ailments included DSD in the form of atypical genitalia, proximal perineal hypospadias and hyperpigmented bifid scrotum, as well as enlarged adrenal glands, elevated ACTH and DHEA-S, albeit without electrolyte imbalance or adrenal insufficiency. This would suggest at least a partial viability of the second allele, however insufficient for a proper virilization of the genitalia during pregnancy. Additionally, the same mutation, also in a heterozygotic variant, has been previously described by Menegatti et al. in two brothers of Italian descent with hypospadias (6).

The final case describes a female infant diagnosed with a homozygous NM_000198.4(*HSD3B2*): c.849delG (p.Trp283fs) variant, causing a frameshift change involving the alteration of a non-conserved nucleotide. The variant allele was found at a frequency of 0.0000041 in 1,461,830 control chromosomes in the GnomAD database, with no homozygous occurrence. Variant has been reported in ClinVar as Likely pathogenic (PVS1, PMS2, PP5). The patient was hospitalized at the age of 4 months due to failure to thrive and was subsequently diagnosed with virilization of the external genitalia, including an enlarged clitoris, severe electrolyte imbalance and adrenal insufficiency, requiring hydrocortisone and fludrocortisone therapy. Additionally enlarged adrenal glands and kidneys with numerous small cysts were reported during early USG examination, as well as recurrent ovarian cysts during subsequent evaluations. Taking into account these symptoms, we theorize that this variant produces an enzyme with a significantly decreased activity. This is corroborated by a theoretical assessment of the mutation effect, which is predicted to cause a stop gain which removes more than 10% of the transcript, critically a section essential to protein function (31).

Thus, the genetic findings in these three children correlate strongly with their clinical presentation, confirming a classic form of 3β-HSD2 deficiency and supporting a clear genotype–phenotype relationship.

### 4.4 Therapeutic aspects

The primary treatment for 3β-HSD2 deficiency involves hormone replacement therapy, with hydrocortisone and fludrocortisone being the most frequently used medications in pediatric patients. In this study, hydrocortisone therapy exhibited both commonalities and variations among the three cases. A shared aspect of treatment was the gradual reduction of hydrocortisone dosage since infancy, with maintenance at 15–40 mg/m²/day in salt wasting cases in early childhood. The dosages were increased during surgeries and illnesses and slightly increased around school age and subsequently stabilized at approximately 10–12 mg/m²/day. However, significant differences were noted in the initial hydrocortisone doses. Cases 1 and 3 required considerably higher starting doses than Case 2. Moreover, Case 1 received substantially higher hydrocortisone doses during infancy than both Cases 2 and 3, primarily due to cardiac complications and the need for preoperative management before cardiac surgery. The suppressed levels of 17OHP and plasma renin activity (PRA) between 4 and 6 months of age, as shown in **Table 2**, indicate overtreatment with both hydrocortisone and fludrocortisone during that period. This indeed reflects a phase of glucocorticoid and mineralocorticoid overdosage rather than optimized therapy. This period of overtreatment likely contributed to the early growth deceleration observed in the patient, as illustrated in **Figure 6**. We acknowledge that, particularly in the early months of life, there was a tendency to use higher doses of hydrocortisone than currently recommended. Based on this experience and current best practices, our approach has since been adjusted to avoid overtreatment and to more carefully titrate glucocorticoid and mineralocorticoid therapy.

In Case 2, the absence of a Synacthen test was a clinical oversight, as such testing would have provided critical information regarding adrenal cortisol reserve. A normal basal cortisol level does not exclude adrenal insufficiency, particularly in the presence of elevated ACTH levels. Cortisol concentrations may appear inappropriately normal or even elevated in congenital adrenal hyperplasia (CAH) due to chronic ACTH stimulation, which does not preclude impaired adrenal reserve.

Early initiation of a low dose of hydrocortisone during the neonatal period might have prevented androgen excess and the subsequent advancement of bone age. Genetic testing should also ideally have been undertaken shortly after the clinical suspicion was raised. The fact that stress dosing was discussed with the parents supports the likelihood of at least partial adrenal insufficiency, even in the absence of overt clinical signs.

The three cases analyzed in this study provided valuable insights into the management of 3β-HSD deficiency. The therapeutic approach shares similarities with that of 21-hydroxylase deficiency. However, in contrast to the treatment for 21-hydroxylase deficiency, the hydrocortisone dosage in 3βHSD2D can be lower, and androgen excess is more easily regulated. It is important to recognize the potential risk of overtreatment in these patients. Excessive doses of hydrocortisone can lead to growth suppression, damage to growth plate cartilage, and cushingoid features, including obesity and metabolic complications such as hypertension, hyperglycemia, dyslipidemia, and reduced bone mineral density. Such as in Case 1 where we observed cushingoid features, short stature and overweight, which are probably linked to very high doses of hydrocortisone (reaching 70 mg/m²/day) required due to cardiac surgery in infancy. Fortunately, this patient additionally presents with a delayed bone age, which gives us hope of adequate growth. On the other hand, Case 3, who was treated with lower hydrocortisone dosage in infancy than Case 1, has appropriate weight for height and does not show cushingoid features, while also suffering from advanced bone age and precocious puberty, which leaves her with a final height drastically below her mid-parental height. This patient showed delayed bone age until the age of 8, when it accelerated, which can be linked to the 2^nd^ stage of thelarche observed at the age of 7 years and 3 months. Taking this into account, if a similar situation is encountered during the treatment of Case 1, additional introduction of a GnRH analogue treatment should be considered. Premature puberty has been previously described in many 3βHSD2D cases [**Table 1** (12, 32–40 **),**, starting as early as at 3 months of age (34). Alos et al. describes a patient with many similarities to Case 3, showing signs of premature pubarche at 4 years of age, accelerated growth and bone age with coexisting bilaterally enlarged ovaries containing multiple cysts, however the study lacks post-pubertal follow-up (32). The authors proposed two possible mechanisms explaining the development of breast and endometrial tissue in this patient. Firstly, they suggest a local conversion of inactive adrenal precursors to estrogens. Secondly, they speculate that pubertal levels of gonadotropins may induce sufficient 3βHSD –activity by increasing the normally low levels of 3βHSD type 1 expression in the ovary, thereby allowing significant ovarian production of estradiol. Some other works also described patients with premature puberty, who additionally displayed advanced bone age (12, 32, 33, 37).

During puberty, our adolescent female patient initially experienced heavy menstrual bleeding and recurrent ovarian cysts, some reaching diameters of up to 50 mm. Similar ovarian cysts have been previously described in female 3βHSD2D patients (32, 41), but there is no data concerning their management. Huang et al. describes a case of recurrent cysts up to 90 mm in size treated with laparoscopic surgery and ovariocentesis (41). This however did not stop the formation of new cysts and soon during an USG examination another one measuring 58 mm was discovered.

In our patient the progression of these cysts was successfully controlled following the introduction of dydrogesterone, a synthetic progestogen with pharmacological properties similar to natural progesterone. It is 10–30 times more potent than oral progesterone, does not cause androgenization or virilization, does not suppress ovulation, and does not elevate basal body temperature. In 3β-HSD2 deficiency, endogenous progesterone production is often inadequate, and 3β-HSD1 activity may be insufficient to compensate. This justifies the use of dydrogesterone from day 16 of the menstrual cycle for 10 days each month. As demonstrated in the **Table 4**, dydrogesterone has been temporarily effective in controlling ovarian cyst formation. However, in our patient, cysts continued to develop alternately in both ovaries, with sizes reaching up to 50 mm. If this condition persists, we plan to initiate treatment with an oral contraceptive pill.

Some studies additionally mention microfollicular ovaries analogous to those seen in the polycystic ovaries syndrome (42, 43). Furthermore, Aslaksen et al. describes the occurrence of premature ovarian insufficiency in a 55-year-old female with Addison’s autoimmune disease and 3βHSD2D (44). Taking all these into account we propose regular gynecological controls with pelvic ultrasound examination.

Another key aspect of 3βHSD2D management in male patients is the treatment of undervirilization. It primarily focuses on addressing underdeveloped male genitalia, including micropenis, hypospadias, and other forms of incomplete masculinization. Surgical correction of hypospadias is generally advised once penile growth has been stimulated, typically between six months and two years of age, a period that aligns with mini-puberty. Case 2 received long-acting testosterone therapy in three doses to promote penile growth, leading to satisfactory penile enlargement and successful recovery following hypospadias repair. This therapy was safe and did not induce GnRH-dependent precocious puberty in our patient. However, at the age of one year and ten months, hydrocortisone was introduced due to increased growth velocity, slight bone age advancement, and suspicion of GnRH-independent precocious puberty. The treatment effectively controlled the condition, maintaining bone age within the normal range.

An additional future concern in our male patient might be adrenal rest tumors (ART), as both testicular rest tumors (TART) (32, 42, 45–50) as well as a case of adrenal rest tumor located in a perineal region have been described **Table 1** **, (** 48). Some of the detected TARTs were found in adulthood and were accompanied by azoospermia (32) or treated with bilateral orchiectomy, due to discomfort and infertility (45), while others were found as early as in the third year of life with coexisting microcalcifications (50). Therefore, we propose a strong focus on regular testicular and abdominal ultrasound examinations as a potential follow-up of male patients with 3βHSD2D since early childhood.

Fludrocortisone dosage is progressively reduced with age, with a recommended maximum dose not exceeding 100 μg/day. Adjustments should be made based on blood pressure, electrolyte levels, and plasma renin activity, ensuring that renin remains within the normal to mid-range during treatment.

Another interesting issue connected with 3βHSD2D management is the connection between autoimmunization and impaired steroidogenesis. We have found a case of coexisting autoimmune Addison’s disease (AAD) and 3βHSD2D, where the authors speculated that there might be other rare unreported cases of autoimmune adrenalitis, due to an early diagnosis of CAH masking the clinical symptoms of AAD (44). Interestingly some rare heterozygous variants in the HSD3B2 gene were found in several AAD patients (44).

### 4.5 Limitations and strengths

This study has certain limitations, primarily the small sample size. It also describes clinical care in a low budget setting, which limits diagnostic procedures, as well as monitoring and treatment options. However, its key strength lies in the detailed presentation of patient management, outlining the challenges encountered and the strategies implemented to address them. The practical value of this work is its most significant contribution.

As a retrospective study, this analysis has greatly enhanced our understanding of the disease. We identified that DHEA-S appears to be a valuable marker for patient monitoring. Morning serum DHEA-S levels—unaffected by circadian variation—proved useful in the follow-up of patients with 3β-HSD2 deficiency. In our setting, the use of age-specific reference ranges has allowed us to rely on this parameter in place of hormones such as ACTH and cortisol which are subject to significant diurnal fluctuations. Furthermore, our review highlighted that the initial doses of hydrocortisone administered were excessively high and may require adjustment in future protocols.

We fully acknowledge, however, that 17-hydroxypregnenolone (17OHPreg) is a more specific and critical marker for biochemical monitoring in 3β-HSD2 deficiency. Unfortunately, this assay is not available in our hospital laboratory due to the very low number of patients and the high cost of testing. From an institutional perspective, implementing this measurement is not considered cost-effective under current conditions. As a result, our monitoring approach is based on routinely available parameters, including serum electrolytes, cortisol (and its daily profiles), ACTH, DHEAS, plasma renin activity, LH, FSH, testosterone, and estradiol. While these tests are not ideal substitutes for 17OHPreg, they allow for reasonable assessment of hormonal control and therapeutic response within the constraints of our clinical setting.

Literature analysis pointed us towards potential follow up targets – TARTs in male patients, bone age assessment and premature puberty, which may require GnRH analogue treatment to prevent final short stature. Only through such a meticulous retrospective evaluation can these errors be recognized and prevented in future clinical practice.

We also acknowledge that the frequency of ultrasonographic assessments in early childhood may have been higher than necessary. Based on our evolving clinical experience, we have since adopted a more conservative and individualized approach, reserving imaging for cases with specific clinical indications rather than routine surveillance.

In our center, surgical correction of severe proximal, midshaft, or distal hypospadias is typically performed within the first year of life, following detailed clinical assessment and written informed consent from the parents. The decision to proceed with early surgical intervention is not based on cosmetic or gender-affirming considerations, but rather on functional and medical indications. These included difficulties with urination due to significant deviation of the urinary stream, increased risk of local skin irritation and inflammation from prolonged urine exposure, elevated risk of urinary tract infections, particularly in severe forms of hypospadias and better healing potential due to more elastic soft tissues in infancy.

We fully acknowledge that surgical timing in DSD and hypospadias is the subject of ongoing international debate and varies by jurisdiction. However, in our practice, early intervention is guided by clinical necessity and parental consent, and is in compliance with national medical and ethical standards.

## 5 Conclusions

This study presents three cases of classic 3β-HSD2 deficiency. All affected infants exhibited genital abnormalities, and disrupted steroid hormone profiles, with the female patients additionally suffering from salt-wasting adrenal insufficiency. The identified mutations were diverse and localized within exon 4 demonstrating a strong genotype–phenotype correlation.

Management primarily involved corticosteroid replacement therapy, which successfully normalized adrenal function. In male patient with hypospadias and micropenis, testosterone administration was used to optimize surgical outcomes for hypospadias repair, rather than delaying intervention until adolescence for penile enlargement. In a female patient with recurrent ovarian cysts dydrogesterone has been used, with limited success, and potential introduction of oral contraceptives has been discussed. Patients also require close bone age and pubertal stage monitoring, in order to ensure proper final height.

The presentation of these cases highlights the necessity of a multidisciplinary approach for managing patients with this steroidogenesis disorder, particularly involving endocrinology, gynecology, and urology specialists. Additionally, complications related to intermittently elevated doses of hydrocortisone and fludrocortisone may affect renal function, leading to hypertension and renal calcifications. Therefore, referral for nephrology and hypertension specialist consultations should be considered in these patients.

## Data Availability

The original contributions presented in the study are included in the article/supplementary material, further inquiries can be directed to the corresponding author/s.
